# Hot Spots of Glacier Mass Balance Variability in Central Asia

**DOI:** 10.1029/2020GL092084

**Published:** 2021-06-09

**Authors:** Martina Barandun, Eric Pohl, Kathrin Naegeli, Robert McNabb, Matthias Huss, Etienne Berthier, Tomas Saks, Martin Hoelzle

**Affiliations:** ^1^ Department of Geosciences University of Fribourg Fribourg Switzerland; ^2^ Laboratory for Environmental Chemistry Paul Scherrer Institute Villigen Switzerland; ^3^ Institute of Geography and Oeschger Center for Climate Change Research University of Bern Bern Switzerland; ^4^ School of Geography and Environmental Sciences Ulster University Coleraine UK; ^5^ Department of Geosciences University of Oslo Oslo Norway; ^6^ Laboratory of Hydraulics, Hydrology and Glaciology (VAW) ETH Zurich Zurich Switzerland; ^7^ Snow and Landscape Research (WSL) Swiss Federal Institute for Forest Birmensdorf Switzerland; ^8^ LEGOS CNRS University of Toulouse Toulouse France

**Keywords:** Tien Shan and Pamir, cryosphere, transient snowlines, annual glacier mass balance time series, climate change, hydrological cycle

## Abstract

The Tien Shan and Pamir mountains host over 28,000 glaciers providing essential water resources for increasing water demand in Central Asia. A disequilibrium between glaciers and climate affects meltwater release to Central Asian rivers, challenging the region's water availability. Previous research has neglected temporal variability. We present glacier mass balance estimates based on transient snowline and geodetic surveys with unprecedented spatiotemporal resolution from 1999/00 to 2017/18. Our results reveal spatiotemporal heterogeneity characterized by two mass balance clusters: (a) positive, low variability, and (b) negative, high variability. This translates into variable glacial meltwater release (≈1–16%) of annual river runoff for two watersheds. Our study reveals more complex climate forcing‐runoff responses and importance of glacial meltwater variability for the region than suggested previously.

## Key Points

- Annual glacier mass balance for Central Asia (1999/00–2017/18) is derived by combining transient snowlines, geodetic surveys, and modeling
- Strong spatiotemporal heterogeneity with contrasting patterns of mass gain and loss are found
- Hot spots of heterogeneous mass balance variability are associated with highly variable glacier melt water runoff

## 1 Introduction

Most glaciers around the world are retreating (IPCC, 2013). During past decades, mass loss has accelerated (Zemp et al., 2019). Glacier responses in High Mountain Asia, including Tien Shan and Pamir, are very heterogeneous spatially (Brun et al., 2017; Farinotti et al., 2015; Kääb et al., 2012; Kraaijenbrink et al., 2017; Scherler et al., 2011; Shean et al., 2020; Wang et al., 2017). Ongoing glacier retreat will have profound consequences on fresh water resources for Central Asia, in particular under high‐emission scenarios (Huss & Hock, 2018; Marzeion et al., 2020; Rounce et al., 2020).

Glacier melt contribution represents up to 40–60% during the late summer months (Aizen et al., 1995, 1997; Armstrong et al., 2019), serving a crucial buffer during droughts (Pohl et al., 2017; Pritchard, 2019). The major Central Asian river basins Syr Darya, Amu Darya, and Tarim will reach maximum glacier meltwater input within the next decades (Huss & Hock, 2018; Rounce et al., 2020). In combination with rapidly growing economies, this poses the risk of freshwater scarcity (Varis, 2014) and might trigger conflicts (Krasznai, 2019; Munia et al., 2016).

The synthesis of regional mass balance dynamics on annual to seasonal time scales for Tien Shan and Pamir is challenging because glaciological data sets are sparse (Barandun et al., 2020; Hoelzle et al., 2019; Unger‐Shayesteh et al., 2013). Therefore, most hydrological models for glacier melt quantification are calibrated on auxiliary data sets rather than actual measurements, hampering their reliability (Pritchard, 2019).

We apply the approach developed in Barandun et al. (2018) for the period 1999/00–2017/18 to all 1,995 glaciers larger than 2 km^2^ (≈60% glacierized area of Tien Shan and Pamir), providing for the first time, an annually resolved and consistent mass balance time series. Our results extend the analysis of spatially heterogeneous glacier responses to the spatiotemporal dimension, and characterize the discharge variability for two medium‐size watersheds in Tien Shan (Naryn River) and Pamir (Gunt River) over the past two decades. This provides first insights into changing year‐to‐year variability in glacier mass change and highlights mountain ranges where important shifts in glacial excess meltwater, i.e., additional water input from glacial water storage reduction (Rounce et al., 2020; Shean et al., 2020) to total river runoff, can be expected.

## 2 Data and Methods

Our methodology combines (i) transient snowlines (transition between ice and snow surfaces approximating the zero‐mass‐balance‐line of the glacier; Dyurgerov et al., 1992), (ii) geodetic estimates, and (iii) distributed mass balance modeling to provide annual mass balance time series (Barandun et al., 2018). The snowlines are used to calibrate a temperature‐index and distributed accumulation model (Braithwaite, 1995) for each glacier and year separately. Geodetic mass balances then constrain the modeled multiyear mass balances to reach agreement between the two observational data sets.

### 2.1 Automatic Snowline Mapping

Over 3,000 Landsat Reflectance Level‐2 science products (Claverie et al., 2015; Masek et al., 2006; Vermote et al., 2016) with cloud cover <50% were collected over ablation seasons (June to September) from 2000 to 2018. We derived spatially distributed shortwave broadband albedo for the glacierized area (Liang, 2001) and removed cloud‐affected pixels (Supplementary Material). We differentiated snow‐covered and bare‐ice surfaces using an automated multistep classification scheme based on the albedo threshold for *certainly snow* (*α* > 0.50) and *certainly ice* (*α* < 0.22) proposed by Naegeli et al. (2019). Ambiguous pixels (0.22 ≤ *α* ≤ 0.50) were evaluated according to their spatial distribution (Supplementary Material). We derived snow‐covered area fractions (SCAFs), i.e., ratio of area above current snowline to total area of the glacier, and filtered for misinterpreted SCAFs according to their seasonal evolution (Supplementary Material). Comparison to manually delineated snowlines in the region result in a root mean square error of <10%.

### 2.2 Geodetic Volume Change

We produced Advanced Spaceborne Thermal Emission and Reflection Radiometer (ASTER)‐derived digital elevation models (DEMs) using MicMacASTER (Girod et al., 2017). We selected 1,201 ASTER DEMs and used 1,852 High Mountain Asia DEMs (Shean, 2017) for differencing based on at least five‐year separation and 40% scene overlap, resulting in 4,243 DEM‐pairs providing data for 902 out of 969 glaciers >2 km^2^ in Tien Shan and 848 out of 1,004 > 2 km^2^ in Pamir. Before differencing, we coregistered DEMs Nuth and Kääb (2011). We calculated ice volume change using a local hypsometric approach (McNabb et al., 2019), converted it into mass change assuming a bulk density *ρ*_Δ*V*_ of 850 kg  m^−3^ (Huss, 2013) and followed the uncertainty calculations in McNabb et al. (2019) for random errors (Supplementary Material).

For comparison with transient snowline‐constrained mass balances, all geodetic mass changes per glacier were homogenized to represent a reference period (1999/00–2017/18). We temporally adjusted geodetic surveys to the common reference period using glaciological measurements (Zemp et al., 2019). Thereby, we calculated the mean annual deviation between each geodetic estimate and the selected glaciological time series over a common time period (Figure S1) and added this deviation to the glaciological measurements for the reference period (Supplementary Material). Two glaciological time series covering the entire study period are available: Tuyuksu and Urumqi. Based on expert knowledge and recent measurements (2010/11–2017/18), we chose Tuyuksu for Dzhungarsky Alatau, Western/Northern Tien Shan, Pamir‐Alay and Western Pamir, and Urumqi series Eastern and Central Tien Shan and Eastern Pamir. All homogenized mean annual mass balances per glacier were weighted according to their uncertainty. The median of all weighted estimates was interpreted as reference geodetic mass balance of the corresponding glacier and compared with literature (Supplementary Material). For glaciers missing geodetic mass balances, the arithmetic mean of all geodetic surveys within the subregion is used.

### 2.3 Transient Snowline‐Constrained Mass Balance Model

We used the model by Barandun et al. (2018) to infer glacier‐specific surface mass balances using the Shuttle Radar Topography Mission (SRTM, Jarvis et al., 2008) DEM for topography and a distributed accumulation and temperature‐index melt model (Braithwaite, 1995) with daily temporal and 30 m spatial resolution. RGI 6.0 outlines (RGI Consortium, 2017) were kept unchanged. Daily total precipitation and 2‐m air temperature from ERA‐interim Reanalysis (0.75° resolution) are used to initiate the model. ERA‐interim was chosen because, unlike other reanalysis products *in situ* observations in mountain regions are assimilated (Orsolini et al., 2019). To calculate melt *M*, a linear relation with positive daily mean air temperature *T*_air_(*x*, *y*, *t*) is applied for each grid cell *x*, *y* and time step *t*

(1) $$M_{x,y,t}=\left\{\begin{array}{cc} {\text{DDF}}_{\text{ice/snow}}\cdot T_{\text{air}(x,y,t)}\; & T_{\text{air}}>0{}^{\circ}\\ 0\; & T_{\text{air}}\leq 0{}^{\circ} \end{array}\right.$$

We use two different degree‐day factors for snow DDF_snow_ and ice DDF_ice_ and at first hold their ratio *R*_DDF_ constant over time.

The snow accumulation *C* is simulated for each grid cell *x*, *y* and *t* by

(2) $$C_{\left(x,y,t\right)}=P_{\text{ERA}}\left(x,y,t\right)\cdot C_{\text{prec}}\cdot \left(1+\left(z_{\left(x,y\right)}-z_{\text{ERA}}\right)\cdot \delta P/\delta z\right).$$

Solid precipitation occurs at *T*_air_ ≤ 1.5 °C with a linear transition range of ±1 °C (Hock, 1999). *P*_ERA_ is the daily precipitation sum of the ERA‐interim grid cell closest to the glacier adjusted to its median elevation *z*(ERA). *z*(*x*, *y*) gives the elevation of each pixel. To correct reanalysis precipitation data to the specific location of each glacier, *P*_ERA_ is scaled with a correction factor *C*_prec_ (Huss et al., 2008, 2009). *C*_prec_ is assumed to be 25% lower for liquid precipitation (Sevruk, 1981). A constant temperature lapse rate *δT*/*δz* and a linear precipitation gradient *δP*/*δz* (Table S2) are applied for extrapolating the temperature and precipitation to each grid cell. Above a critical elevation *Z*_crit_ precipitation is held constant. The constant model parameters (Table S2) are based on Barandun et al. (2018).

For a first‐order calibration, we use SCAFs to constrain model parameters for accumulation (*C*_prec_) and melt (DDF_snow_) for each glacier and year (Supplementary Material). Barandun et al. (2018)'s calibration procedure is adjusted to limit computation time. A start value for DDF_snow_ and *C*_prec_ is iteratively narrowed down until no improvement of the model is observed (Figures S6 and S7). Barandun et al. (2018) found an uncertainty of ≈0.10 m w.e.  yr^−1^ related to an overestimation and underestimation of the mapped snowlines, showing the model sensitivity to a single snowline delineation to be relatively small, provided that enough and temporally well‐distributed snowline observations are available.

*C*_prec_ and DDF_snow_ are calibrated annually and for each glacier separately to correctly represent winter snow accumulation and melt rates for each year (Figures S6 and S8, Barandun et al., 2018). The model is run from 1999/00 to 2017/18 for every year with at least two available snowline observations (Barandun et al., 2018). Calibrated parameters obtained for years with good data availability were averaged for remaining years. One hundred and thirteen glaciers had fewer than 16 out of 18 years with sufficient snowline maps and were removed.

In contrast to conventional modeling, our results are tied to subseasonal snowline observations for each glacier and year, as well as multiannual geodetic surveys. This reduces the model sensitivity to input variables (Barandun et al., 2018). Using transient snowline observations for model calibration reproduced year‐to‐year mass balance variability close to observations (Table S3), improving conventional modeling.

For the second‐order calibration, we constrain annual mass balances by comparison with geodetic surveys of each glacier for the reference period. The relation between initially constant degree‐day factors of snow and ice *R*_DDF_ (first‐order calibration) are adjusted within a plausible range (1.2–2.9, Hock, 2003), and then the first‐order calibration is repeated (Figure S6) in case the snowline‐constrained mass balance series exceeds the error range of the geodetic approach. When no agreement is found but the plausibility limit for *R*_DDF_ reached, the precipitation gradient (*δP*/*δz*) is adjusted within realistic bounds (0.5–20% 100 m^−1^; Immerzeel et al., 2015) and previous calibration steps repeated. These literature‐based parameters are spatially highly variable and not well constrained for individual glaciers. The final parameter range is summarized in Figure S8 and Table S2.

The second‐order calibration is repeated until the absolute difference between the two approaches is smaller than the uncertainty of the geodetic method or both parameters reach their plausibility limits. After this step, 283 (14%) glaciers were omitted. Finally, we filtered the time series for outliers by removing all annual mass balances unrealistically high or low (two standard deviations beyond the mean, 377 glaciers removed) and only include the resulting series if still in agreement with the geodetic survey.

We adopt the mean uncertainties (±0.32 m w.e. yr^−1^) associated with the snowline‐constrained mass balance model from Barandun et al. (2018) and combine it with the error estimate from the geodetic surveys. This conservative estimate of ±0.37 m w.e. yr^−1^ does not assume independence of the errors from year to year.

### 2.4 Glacier Meltwater Excess

**Figure 1:**
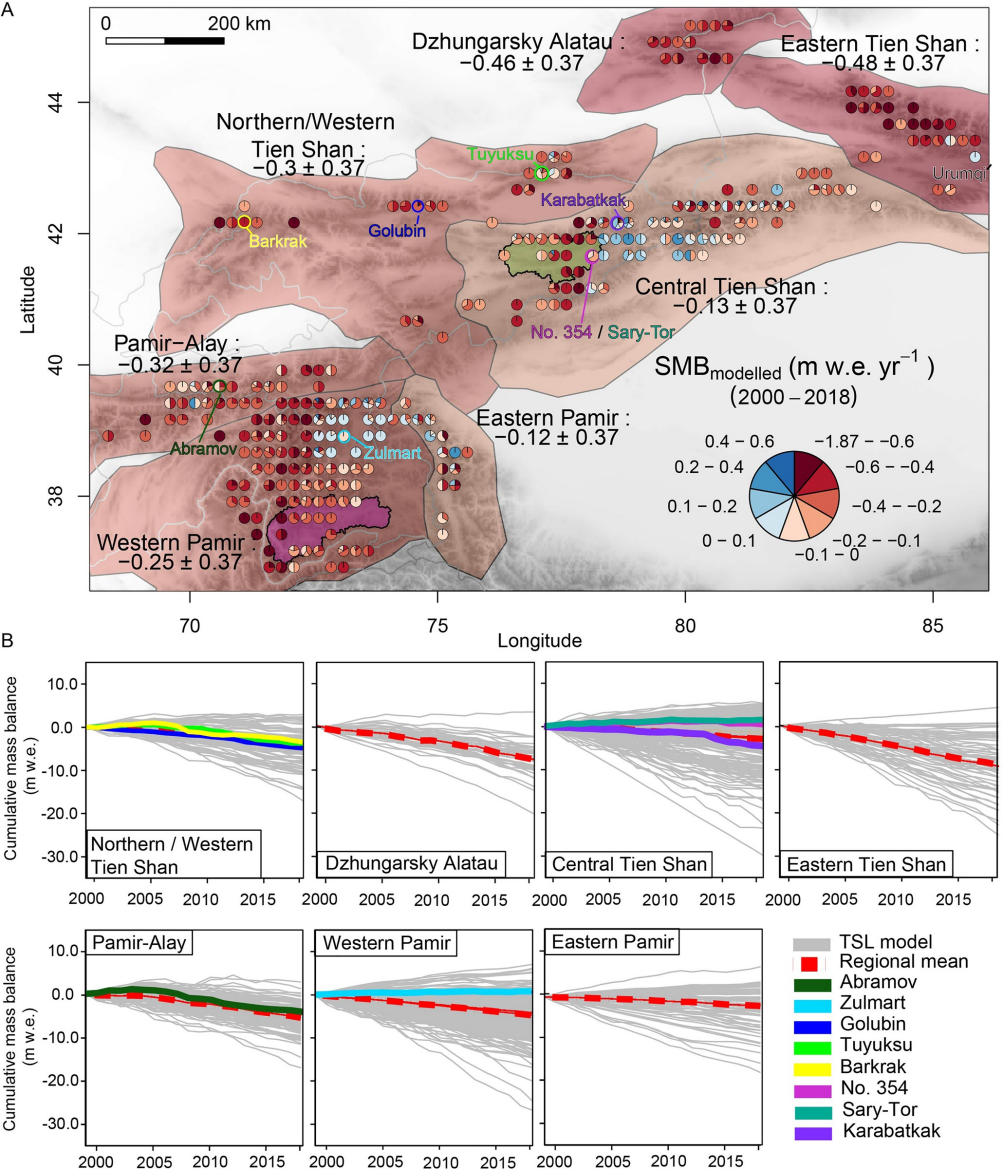
(a) Mean annual mass balances (1999/00–2017/18) for different subregions. Pie slice sizes representing percentage of glaciers in each category (binned to 0.25‐degree grid cells using glacier centroids provided in the RGI) not scaled to total number of glaciers per grid cell (Figure S11: pies scaled to number of glaciers on 0.75° ERA‐Interim grid). Regional mass balances are area‐weighted means of glacier values. Colored circles indicating location of monitored glaciers. Magenta polygon showing Gunt (Western Pamir) catchment, and green polygon Naryn River (Central Tien Shan) catchment. (b) Reconstructed cumulative mass balance series (gray lines) compared with regional mean (red dashed lines) and reconstructed mass balances of monitored glaciers (colored continuous lines) per subregion.

The effect of glacier mass balance on river discharge is assessed using two discharge data sets of Naryn (Tien Shan) and Gunt (Pamir) rivers (Figure 1a). For each catchment, three monthly time series were obtained (Table S4): long‐term average discharge, one extremely negative (2009 for Gunt, 2003 for Naryn) and one positive (2008 for Gunt, 2006 for Naryn) mass balance year. The long‐term discharge is the periods 1999/00–2017/18 (Naryn) and 1999/00–2012/13 (Gunt). The Naryn and Gunt catchments have 8% and 5% glacier cover. Discharge estimates are based on annually calibrated stage‐discharge rating curves and stage readings. Both catchments drain higher parts of the respective mountain ranges so that runoff is not impacted by water resource management (e.g., dams, agriculture).

We calculate excess glacier meltwater contributions to river runoff due to decreasing water storage for negative glacier mass balances according to Shean et al. (2020) at annual time scale, and at monthly time scale for some extreme years to highlight strong variability. All annual mass balances >0 m w.e. yr^−1^ are set to zero, and mass balances of all glaciers <2 km^2^ and nonmodeled glaciers are assigned the mean mass balance of the catchment (Shean et al., 2020). The smallest glaciers (<0.2 km^2^), which might deviate strongly from this assumption (Shean et al., 2020), make up 9% and 7% of the total glacierized area in the Gunt and Naryn catchments, respectively, and contribute little to the overall uncertainty. However, we stress that our results are a first‐order estimate. We use the monthly glacier mass change constrained with the transient snowline observations during the summer season (June to September) to calculate the monthly anomalies for a very negative and very positive mass balance year from the average monthly mass balance for each catchment. We assume mass balance and glacier melt to scale linearly (Gao et al., 2010).

## 3 Annual Glacier Mass Balance for the Tien Shan and Pamir

Our mass balance calculations, tied to transient snowline observations, provided annual values for 1,222 (61%) glaciers >2 km^2^ (Table S5). The remaining 773 (39%) were excluded due to insufficient transient snowline observations or disagreement with geodetic surveys.

According to our multidata assessment, we found an area‐weighted average glacier mass balance of −0.23 ± 0.37 m water equivalent (w.e.) yr^−1^ from 1999/00 to 2017/18 for Tien Shan and Pamir. Glaciers in Eastern Tien Shan and Dzhungarsky Alatau showed the most negative mass balance rates at almost −0.50 ± 0.37 m w.e. yr^−1^ (Figure 1a). Glaciers in Northern/Western Tien Shan, Pamir‐Alay, and Western Pamir showed mass balance rates between −0.25 ± 0.37 and −0.32 ± 0.37 m w.e. yr^−1^. A moderately negative mass balance rate of roughly −0.13 ± 0.37 m w.e. yr^−1^ was observed for Central Tien Shan and Eastern Pamir. Spatially contrasting mass balances within the subregions, and partly for glaciers in close vicinity, challenge regional averaging, and question existing standard regional divisions (Figure 1a).

Our results highlight that several glaciers of the existing international monitoring network represent the regional averages fairly well (Figure 1b) but some deviate. The observation network is so far unable to capture the spread within their subregions (e.g., Western Pamir; Figure S9) or no observations at all are available (e.g., Dzhungarsky Alatau).

## 4 Spatiotemporally Heterogeneous Glacier Response

**Figure 2:**
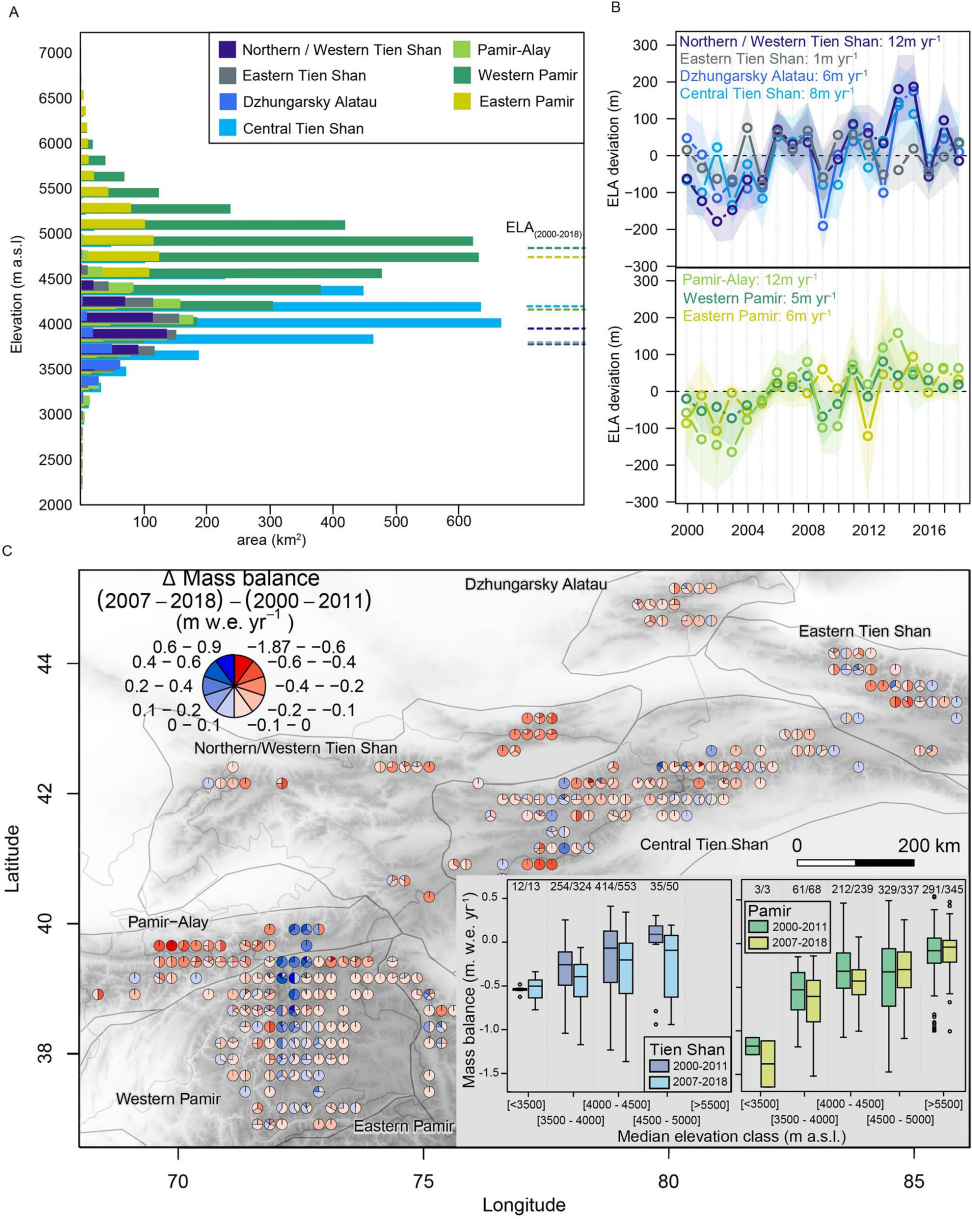
(a) Area‐elevation distribution (Bolch et al., 2019) and mean reconstructed ELA (dashed lines) per subregion (1999/00–2017/18). (b) Annual ELA anomalies per subregion for Tien Shan (blue colors) and Pamir (green colors). (c) Pie charts showing difference in mass balance between second (2006/07–2017/18) and first (1999/00–2010/11) period binned in 0.25‐degree grid cells. Pie slice sizes representing percentage of glaciers in given class of mass balance change (*δ* mass balance). Boxplots in inset show mean annual mass balances per median elevation class for both periods. Top of boxes numbers indicate number of considered glaciers versus total number of glaciers per class according to RGI 6.0. ELA, equilibrium‐line‐altitude.

From 1999/00 to 2017/18, we found negative trends in annual mass balance for Tien Shan, significant for Northern/Western and Central Tien Shan (*P*‐value <0.05) but not for Eastern Tien Shan and Dzhungarsky Alatau. No clear temporal trend over this period was found for Western Pamir, but significant negative trends were observed for Pamir‐Alay and Eastern Pamir. While at the southeastern part of Tien Shan and Pamir over 60% of the glacierized area remained above the equilibrium‐line‐altitude (ELA), <40% did at the northwestern margin, suggesting substantial glacier mass loss under current climatic conditions for the latter (Figure 2a).

Prior to 2005, many glaciers had close‐to‐zero or slightly positive mass balances and ELAs below the 1999/00–2017/18 average (Figures S10 and 2b). Subsequently, mass balance became more negative, and ELA increased gradually in most subregions until 2018. In Eastern Tien Shan and Western Pamir, the ELA remained close to the average from 1999/00 to 2017/18, and only nonsignificant negative trends in mass balance were revealed. “Hot spots” of nearly balanced conditions in Western Pamir contrasted with a significant negative mass balance in Eastern Tien Shan (Figure S10). A similar “hot spot” was found in Central Tien Shan. Eastern Tien Shan was the only region where the ELA stayed below median glacier elevations (Table S5).

Previous studies (Brun et al., 2017, 2019; Shean et al., 2020) highlighted spatially heterogeneous glacier responses of High Mountain Asia. Our results show this heterogeneity to be pronounced down to subregion‐scale of Tien Shan and Pamir, revealing heterogeneous mass changes over time (Figure 2c). Between a first (1999/00–2010/11) and a second (2006/07–2017/18) period chosen to overlap to avoid leverage effects of potential extreme years, overall mass balances became more negative. However, glaciers with less negative mass balance for the second period are apparent, often where mass loss from 1999/00 to 2017/18 was high (Figure 2c).

In the three subregions of Pamir, glaciers with low median elevations (<4,500 m a.s.l.) experienced accelerated mass loss in the second period (Figure 2c). With higher median elevations (i.e., >4,500 m a.s.l.), the signal weakened or even inverted to unchanged or slightly positive mass changes. Considering 500 m elevation bins for Tien Shan, almost all elevation classes had more negative mass balances for the second period (inset Figure 2c).

**Figure 3:**
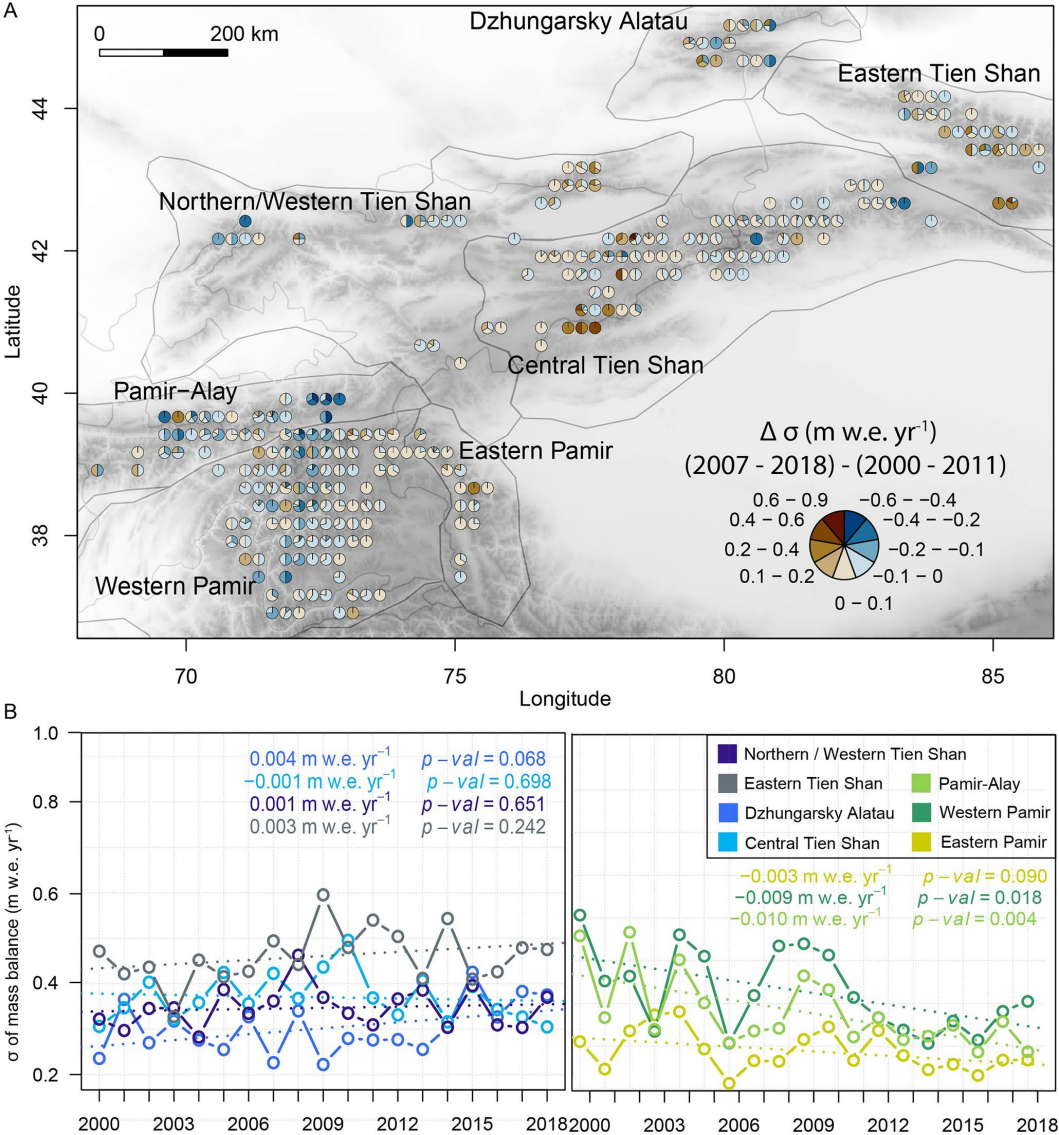
(a) Differences in mean standard deviation (*σ*) between periods 2006/07–2017/18 and 1999/00–2010/11 for each glacier binned for 0.25‐degree grid cells. Pie slice sizes representing percentage of glaciers in a *σ* change category. (b) Mean annual *σ* for all glaciers within a subregion (1999/00–2017/18) for Tien Shan (blue) and Pamir (green).

The spread in glacier mass balances was generally similar or higher within the different classes for Tien Shan subregions, while generally decreased within individual classes in Pamir for the second period (Figure 2c). A declining regional variability (i.e., mean of the standard deviation of all mass balances within a region per year for either time period) in Pamir (Figure 3b) adds to a spatially homogeneous character. In contrast, increased spatiotemporal heterogeneity is present for Tien Shan from 1999/00 to 2017/18 (Figures 3a and 3b).

## 5 Changing Mass Balance Variability

Typically, Tien Shan and Pamir are classified into subcontinental (i.e., Western/Northern Tien Shan, Pamir‐Alay) to continental (i.e., Central Tien Shan, Western and Eastern Pamir) regimes (Wang et al., 2019). We observed lower annual mass balance variability for more continental parts of Central Tien Shan and Central and Eastern Pamir. Higher variability dominates toward the subcontinental western margin, generally accompanied with higher mass loss (Figures S12 and 1a). We found lowest year‐to‐year variability for Central Tien Shan (standard deviation *σ* = 0.29 m w.e.) and for Western and Eastern Pamir (*σ* = 0.29 and 0.21 m w.e., respectively) (Figure S12). These high‐elevation continental glaciers (Table S5) previously showed higher sensitivity to precipitation changes and nonuniform mass balance sensitivity with elevation (Wang et al., 2019). Low mass balance sensitivity and variability and more gentle mass balance gradients relate typically to dryer and colder environments (Oerlemans, 2001). Within more subcontinental subregions, a higher year‐to‐year variability (greater *σ* = 0.39 m w.e., Figure S12) is in agreement with previously shown elevated sensitivity to changing atmospheric conditions, especially air temperature (Wang et al., 2019). Stark contrasts within the subregions, especially for Western Pamir and Central Tien Shan, show that the “hot spots” of spatially heterogeneous glacier response (Figure 1) are accompanied by a strong year‐to‐year variability (Figure S12) and correspond only partially to proposed regional continental/subcontinental classifications.

We compared the standard deviations in annual mass balances (Figure 3a) between the periods of 1999/00–2010/11 and 2006/07–2017/18. In Tien Shan, increasingly negative mass balances tend to be mirrored in slightly increased annual variability (Δ*σ*: 0.03 m w.e. yr^−1^). However, some areas in Central Tien Shan with high mass loss rates showed less negative mass balances but increased variability in the 2007–2018 period (Figures 3b and 2c). Changes in Pamir are also diverse (Figure 2c). For example, the year‐to‐year variability strongly decreased in parts of Western Pamir, where mass balances were less negative for the second period (Figure 3a). Our data show that a decrease in mass balances from 1999/00 to 2010/11 and 2006/07 to 2017/18 was not always accompanied with an increase in year‐to‐year variability and vice versa (Figure 2c). Still, areas with the smallest reduction in year‐to‐year variability were generally those with more positive mass balances from 1999/00 to 2017/18, and largest changes in variability were related to more pronounced mass loss.

Anticipated climate change in Tien Shan and Pamir (Aizen et al., 1997; Haag et al., 2019) is expected to continuously alter glacier sensitivities to air temperature and precipitation (Dyurgerov et al., 1994) and enhance spatiotemporal heterogeneity. Despite local differences, mass balance variability is on average higher (*σ* = 0.43 m w.e. yr^−1^) for glaciers with more negative mass balances than for glaciers with above‐average mass balances (*σ* = 0.15 m w.e. yr^−1^). Under ongoing climate change, mass balances are expected to become increasingly negative and more variable in space and time, ultimately increasing the variability of glacier meltwater contributions with more pronounced extremes and changes in glacier runoff dynamics with important socio‐hydrological consequences in the future (Nüsser, 2017).

Climatic forcing has been previously identified as dominant driver for the heterogeneous mass balance sensitivity over High Mountain Asia, explaining up to 60% of its spatially contrasting glacier response (Sakai & Fujita, 2017). Glacier morphology was found to explain up to 36% of the spatial mass balance variability for Tien Shan and 20% for Pamir‐Alay, but only 8% for Western and Eastern Pamir (Brun et al., 2019). The influence of glacier surge activity is so far poorly understood (Goerlich et al., 2020). Although our results provide new estimates to revisit these analyses, prevailing large uncertainties in current meteorological data sets (Zandler et al., 2019) require a more detailed approach than can be provided here.

## 6 Relevance of Glacier Mass Loss to River Runoff

The most important impact of changing glacier runoff dynamics for Central Asia is the uncertain timing and duration of high or low meltwater contributions to river systems during dry summer months (Chen et al., 2018; Immerzeel et al., 2020; Varis, 2014). Glacier melt can mitigate extreme water shortages on seasonal to decadal time scales (Pohl et al., 2017; Pritchard, 2019).

**Figure 4:**
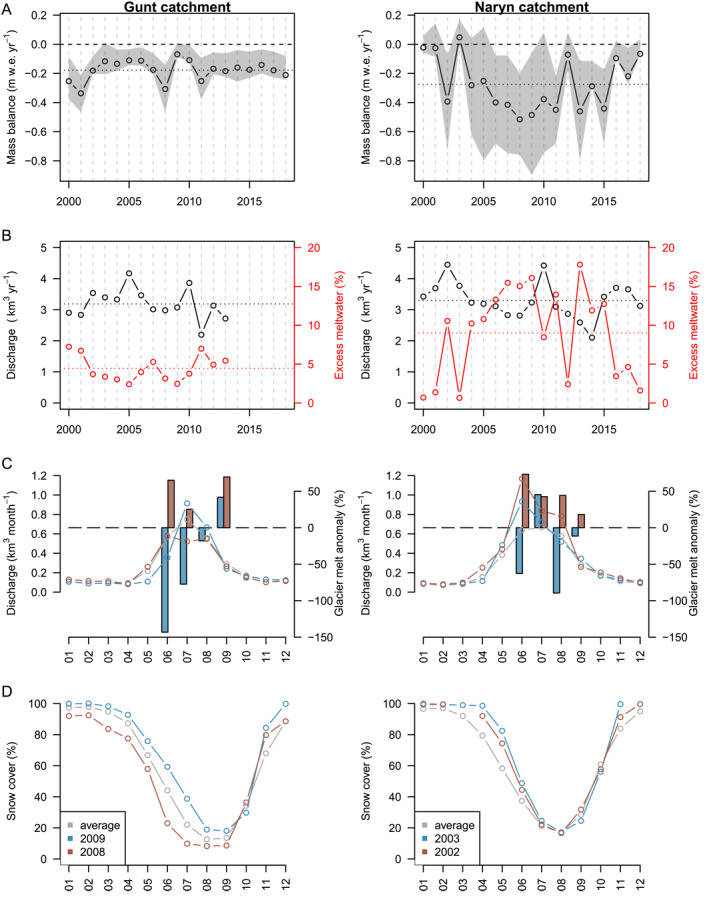
(a) Mean annual mass balances for Gunt and Naryn catchments (1999/00–2017/18). Shaded gray area indicating spread between individual glaciers within a catchment. (b) Annual river discharge measured (black) in comparison to excess glacier meltwater runoff contribution (red) to total river discharge. (c) Monthly discharge (lines) as long‐term average (1999/00–2017/18, gray), for below‐average (red: 2002/03, 2008/09), and above‐average (blue: 2001/02, 2007/08) mass balance years. Monthly melt anomalies with respect to average melt for each month (bars). Melt anomalies lower than −100% representing positive mass balances. (d) Monthly snow‐cover from MODIS10CM (Hall & Riggs, 2015) for both catchments.

The monthly observations of the summer (Naryn) and winter (Gunt) precipitation‐dominated catchment (Figures 1 and S13; Table S4) provide insights for two representative watersheds for the headwaters of Central Tien Shan (Aizen et al., 1995) and Pamir (Pohl et al., 2017). The average additional water released due to annual excess glacier melt runoff is ≈9% for Naryn and ≈5% for Gunt. The annual excess meltwater contribution increases to ≈16% and ≈7% for most negative mass balance years (Figures 4a and 4b). Despite a relatively low annual contribution, melt water excess can become crucial during dry summer months (Armstrong et al., 2019; Barandun et al., 2020; Pritchard, 2019). Glacier melt from June to September can increase by ≈90% (Naryn) and ≈40% (Gunt) above average monthly melt production during an extreme negative mass balance year (Naryn: 2001/02; Gunt: 2007/08, Figure 4c). The largest relative increases in glacier melt are observed in June due to differences in snow‐cover depletion, particularly pronounced for Gunt (Figures 4c, 4d, and S13). For Naryn, the importance of glacier melt in August 2002 is almost doubled compared with an average year, probably related to increased air temperatures (Figure S13). Summer glacier melt for very negative mass balance years can be twice as high as for extremely positive mass balance years (e.g., 2002/03, 2008/09), when meltwater production decreases by 20–50%. Highly glacierized catchments (10–30%) show larger compensating effects in Pamir, i.e., lack in precipitation and subsequent snow‐cover results in higher glacier melt contribution to total runoff, and vice versa (Pohl et al., 2017). However, for the Gunt catchment, containing less glacierized area, a direct response of discharge to snow‐cover changes is revealed (Figures 4b and 4e).

Increasing year‐to‐year mass balance variability profoundly influences the excess glacier meltwater contribution to total river runoff at the end of summer. This might become especially visible for dry periods with reduced snow‐cover (Aizen et al., 1995, 2007). We expect the most precarious changes in fresh water release variability through glacier melt for highly glaciated, subcontinental mountain ranges [e.g., Dzhungarsky Alatau, Western/Northern Tien Shan, Pamir‐Alay], where strongly negative mass balances are associated with large increasing year‐to‐year variability (Figure 3).

## 7 Conclusions

We provide annual mass balance time series with low sensitivity to meteorological input, closely tied to transient snowline and multiannual geodetic surveys for the data‐sparse mountain ranges in Central Asia. Our results show strong variability in mass balance between successive years across Tien Shan and Pamir. The transient snowline approach reveals spatiotemporal heterogeneity in which positive and negative glacier mass balances stand in stark contrast. These “hot spots” extend the Karakorum anomaly to other subregions in Central Asia. While our findings show signs of spatiotemporally more homogeneous glacier responses in Pamir, glacier mass balances in Tien Shan have become increasingly heterogeneous. The derived clusters and the annually resolved mass balances provide a new basis to investigate possible importance of meteorological and morphological drivers and their variability in unprecedented temporal and spatial resolution.

The excess glacier meltwater release for two catchments representative for Central Asia can highly vary between years (1–16%). The difference in meltwater production can relate strongly to early summer snow depletion (up to six times more melt than average) or follow air temperature changes during the end of summer (up to three times more melt than average).

Large uncertainties in meteorological data sets remain a major hurdle for our understanding of processes governing changes at the climate‐glacier‐runoff nexus. Ongoing monitoring efforts coupled with mass balance and runoff models are essential to understand the impact of climate upon future glacier mass balance and discharge patterns, and in turn, to develop policy responses to rapidly increasing water demands in Central Asia.

## Supporting information

Supporting Information S1

## Data Availability

ERA‐Interim Reanalysis data are available at https://apps.ecmwf.int/datasets/data/interim-full-daily/ (last access: 11.12.2020). Landsat imagery and SRTM are available at https://earthexplorer.usgs.gov/ (last access: 11.12.2020). High Mountain Asia DEMs are available at https://nsidc.org/the-drift/data-set/hma/ (last access: 11.12.2020). ASTER imagery is available at https://earthdata.nasa.gov/ (last access: 11.12.2020). MODIS MOD10CM data are available at https://nsidc.org/data/mod10cm (last access: 11.12.2020). High Asia Refined Reanalysis data are available at https://www.klima.tu-berlin.de/HAR (last access: 11.12.2020). Discharge data used in this study are available in the Supplementary Material of the study. These data were obtained from the Tajik Hydromet http://www.meteo.tj/ as part of the BMBF (Federal Ministry of Education and Research) research programme PAMIR (FKZ 03G0815) for the Gunt River, and from the Kyrgyz Hydromet http://meteo.kg for the Naryn River. Due to the hydromet institutions' data policies, the data are not readily available for download from the respective websites but can be requested through the contact links. Annual mass balance time series are provided via zenedo open‐access repository (https://doi.org/10.5281/zenodo.4782116).
