# Supplementary material for: Hot Spots of Glacier Mass Balance Variability in Central Asia
Source: Geophys Res Lett. 2021 Jun 9;48(11):e2020GL092084. doi: 10.1029/2020GL092084 (PMC8244088; doi:10.1029/2020GL092084)
Supplement: Supplementary file 1 — Supporting Information S1 [file GRL-48-e2020GL092084-s001.pdf]

Supporting Information for "Hot spots of glacier mass balance variability in Central Asia"

Martina Barandun[1,2], Eric Pohl[1], Kathrin Naegeli[3], Robert McNabb[4,5],

Matthias Huss[1,6,7], Etienne Berthier[8], Tomas Saks[1], Martin Hoelzle[1]

[1]Department of Geosciences, University of affiliation, Fribourg, Switzerland

[2]Laboratory for Environmental Chemistry, Paul affiliation Institute, Villigen, Switzerland

[3]Institute of Geography and Oeschger Center for Climate Change Research,University of Bern, Bern, Switzerland

[4]School of Geography and Environmental Sciences, Ulster University, Coleraine, United Kingdom

[5]Department of Geosciences, University of Oslo, Oslo, Norway

[6]Laboratory of Hydraulics, Hydrology and Glaciology (VAW), ETH Zurich, Zurich, Switzerland

[7]Snow and Landscape Research (WSL), Swiss Federal Institute for Forest, Birmensdorf, Switzerland

[8]LEGOS CNRS, University of Toulouse, Toulouse, France

Contents of this file

1. Methodological details
2. Limitations
3. Figures S1 to S13
4. Tables S1 to S5

Methodological details

Automatic transient snowline mapping

Transient snowlines were mapped automatically on the surface reflectance products of Landsat Thematic Mapper (TM) / Enhanced Thematic Mapper Plus (ETM+) and Operational Land Imager (OLI). The scenes consist of six (TM/ETM+) or seven (OLI) individual spectral bands in the wavelength range of around 440 nm to 2300 nm, with slight deviations of the individual band widths for the specific sensors. They were atmospherically corrected but lack a correction for topographic or shadow effects. More detailed information about these level-2 science products can be found in the product guides provided by the U.S. Geological Survey and in the literature (Masek et al., 2006; Claverie et al., 2015; Vermote et al., 2016). Thereby, we derived spatially distributed shortwave broadband albedo for the glacierised area of each image. From the maps we discriminated snow-covered and bare-ice surfaces using an automated, multi-step classification scheme after Naegeli et al. (2019).

Before applying the classification algorithm, we performed two preparatory steps. First, clouds were detected and removed. For this, the Spectral Angle Mapper (SAM) classification algorithm implemented in ENVI with manually derived, sensor-specific spectral libraries for different cloud signatures was used for cloud detection (Kruse et al., 1993; Naegeli et al., 2019). For each scene a cloud mask was obtained that was used to exclude cloud-affected pixels from all consecutive analyses. Simultaneously, Landsat 7 SLC-off void-stripes were filtered out. We did not remove oversaturated pixels of the Landsat scenes. After visual inspection, pixel saturation was observed mainly on TM/ETM+ images and predominately in the snow covered area. These pixels result in unrealistically

high albedo values (>1.00). Using the here applied classification scheme, oversaturated areas will be classified as snow and hence do not affect the snowline classification under the assumption that for the target regions and scenes such oversaturated pixel concerned only snow. In a second step, we applied the narrow-to-broadband conversion by Liang (2001) to obtain shortwave broadband albedo from the individual spectral bands. The conversion was based on five of the seven individual bands. For Landsat OLIs, the band numbers were adjusted accordingly (Naegeli et al., 2017; Naegeli & Huss, 2017). Naegeli et al. (2017) demonstrated the high accuracy of such albedo products.

The most important classification steps are summarised hereinafter and more methodological details can be found in Naegeli et al. (2019). We used the RGI outlines to extract the albedo maps for each glacier larger than 2 km^2 on available Landsat scene for the Tien Shan and Pamir. The glacier area limit of 2 km^2 is related to the capability of transient snowline detection on Landsat satellite images, and was chosen based on a visual check on manual mapping. Each glacier-specific map with less than 20% information content above and below the median glacier elevation was removed. We defined two threshold values for *certainly snow* (α >0.50) and *certainly ice* (α <0.22) based on literature values (Cuffey & Paterson, 2010). Critical albedos, for which an unambiguous assignment of the surface type was not possible, vary between a value of 0.22 and 0.50. Values within this range can hence belong to either snow or ice surface type. We evaluated the ambiguous pixels according to their spatial distribution over the glacier area. For this, we calculated the average albedo in each 30 m-elevation band where we encountered critical albedo pixels,

and traced the albedo change with elevation. The greatest slope on the albedo-elevation profile defined the critical albedo (α_{crit}), implying the limit between snow and ice / firn, thus the transient snowline. The critical albedo was considered to be a site- and scene-specific albedo threshold (α_{crit}) and was used as reference value to re-evaluate the pixels within the range of critical albedo values of the specific scene. In order to reclassify extreme outliers, all grid cells were re-examined regarding their relative position compared to the beforehand defined snowline. We used a probability-based approach: an increasing positive / negative vertical distance from the transient snowline resulted in a decrease in likelihood of the cell to associate to the class on opposite site of the transient snowline. Pixels were reclassified accordingly.

We derived the snow-covered area fraction (SCAF) i.e. the ratio of the area above the current snowline to the total area of the glacier, to include the glacier-specific hypsometry. Glacier outlines were taken from Randolph Glacier Inventory version 6 (RGI 6.0, RGI Consortium (2017)). Additionally, to avoid misclassification of completely snow-covered glacier surfaces, we assumed that the median albedo within the lower quarter of the glacier only exceeded the value of >0.35 in the case of an entirely snow-covered glacier. In a last step, the transient snowlines used for model calibration were filtered to avoid misclassification due to sporadic fresh snowfall based on the temporal evolution of the snowline throughout the ablation season. The minimum observed snow covered area fraction (SCAF) at the end of the summer defined the end of the ablation season, and all following SCAFs were omitted.

The separation of snow and ice primarily depends on the albedo of the surface classes. It is possible to account for fresh snowfall or changes in other surface characteristics with a physical interpretation of this value. However, the range of albedo for snow and ice / firn can overlap considerably, and a straightforward classification is not always possible (Naegeli et al., 2019). Nonetheless, the transition of the albedo between ice and snow is spatially characterised by a distinct change (Hall et al., 1987), and the greatest slope of the albedo-elevation profile approximates the limits between snow and bare-ice surfaces well (Naegeli et al., 2019). On average, 5-10 SCAFs are available per ablation season and glacier.

Comparison to manually delineated snowlines for Abramov, Golubin and Glacier No. 354 showed satisfying agreement within an RMSE of less than 10% for all mutually observed SCAFs. However, the validation focused on clear-sky images. The applied cloud mask using SAM does not filter very thin optical clouds and hence can interfere with automated surface classification.

Misinterpretation of the snowline on the satellite images can affect the model performance and a thorough filtering of snowlines is recommended. Barandun et al. (2018) found an uncertainty of $\approx 0.10\,\mathrm{m\,w.e.\,yr^{-1}}$ related to a systematic over- and underestimation of the mapped snowlines and showed that the model sensitivity to single snowline delineation is not strong if enough and well-distributed information on the transient snowline position is available. This highlights the benefit of the used method to not solely depend on the end-of-summer snowline as a proxy for the surface mass balance. Including all available

transient snowline observations hence helps to guarantee a robust calibration procedure.

Geodetic volume change

We produced ASTER DEMs using the MMASTER processing chain (Girod et al., 2017). To remove biases due to sensor motion in the ASTER DEMs, we used the SRTM DEM as a reference, as it provides a consistent DEM for the entire region. We then masked the final DEMs using the correlation score provided by the MMASTER processing, using a threshold for the correlation score of 70%.

To filter the DEM differencing pairs, we employ a filtering procedure, modified after Pieczonka and Bolch (2015), to remove remaining outliers on the glacier area. The introduced filter used the overall standard deviation (σ) of the glacier elevation difference, weighted by an elevation-dependent coefficient, allowing both positive and negative elevation changes in the ablation and accumulation area. The weighted coefficient for each pixel was determined using a sigmoid function. After a normalisation of all pixels on the glacier surface to its elevation range (Eq. 1), a weighting coefficient c was calculated using the normalised glacier elevation w (Eq. 2). This coefficient was then multiplied with the standard deviation of the glacier elevation change σ_{dh} to indicate the expected maximum change of each pixel on the glacier surface δh_{max}.

$$w = \frac{z_{x,y} - z_{min}}{z_{max} - z_{min}} \tag{1}$$

where $z_{x,y}$ is the pixel on the glacier surface, z_{min} and z_{max} the minimum and maximum glacier elevation, respectively.

$$c = \frac{1}{1 - tanh(\frac{\pi}{2} - 3w)} \tag{2}$$

$$\delta h_{max} = c \times \sigma_{\mathrm{dh}} \tag{3}$$

Because of low correlation due to clouds, shadow, or bright snow, the DEMs (and therefore the elevation change maps) had significant voids over the glaciers, which prevented a straightforward summation of the elevation differences to calculate volume changes. Therefore, we used the local mean hypsometric approach (McNabb et al., 2019) to calculate volume changes. First, we estimated the area-altitude distribution for each glacier based on the SRTM DEM, using either 50 m elevation bins or bins corresponding to 10% of the glacier elevation range, whichever was smaller. For each DEM differencing scene, we then took the mean elevation difference per elevation bin, iteratively removing remaining outliers that were more than three standard deviations away from the mean of the bin, which yielded an estimation of the elevation difference as a function of elevation. We then removed all values where the bin was more than 40% void (i.e., no data available). If the elevation curve still covered at least 75% of the glacier area based on the area-altitude distribution, the curve was filled using linear interpolation, setting elevation difference values above and below the glacier elevation range to zero. Elevation difference maps where less than 50% of the glacier area was sampled or the root mean square of the elevation difference on stable ground was more than 10 m were removed. Finally, we calculated

the volume change as the sum of the product of the elevation difference curve and the area-altitude distribution. We followed the uncertainty calculations proposed by McNabb et al. (2019) to estimate the random error of all inferred mass changes. The calculated uncertainty estimate included three components: (1) the uncertainty related to the elevation change observed over stable ground, (2) the uncertainty of elevation change determination over glacierised areas and (3) the uncertainty in the volume-to-mass conversion.

The difference in volume changes ΔV for each glacier based on ASTER scenes were converted into geodetic mass balances ΔM_{geod}, using a density $\rho_{\Delta \mathrm{V}}$ of $850\,\mathrm{kg\,m^{-3}}$ (Huss, 2013):

$$\Delta M_{\mathrm{geod}} = \frac{\Delta V \cdot \rho_{\Delta \mathrm{V}}}{\overline{A} \cdot \Delta t} \quad (4)$$

where, $\overline{A}$ is the glacier area and Δt is the time in years between the corresponding image pairs.

Homogenisation of geodetic mass balances

A large amount of overlapping ASTER scenes and HMA DEM for the studied glaciers led to a range of different geodetic mass balances with different time stamps per glacier (S1). In order to provide a suitable and robust result for a second-order calibration all geodetic mass changes for each individual glacier i were homogenised to represent a fixed reference period t_{ref} (1999/00 to 2017/18). To homogenise geodetic mass balances covering arbitrary periods t to the reference period we followed Zemp et al. (2019). First, we calculated the mean annual deviation $\overline{\beta_{\mathrm{t}}}$ between each geodetic estimate M_{geod} and

the selected glaciological time series M_{glac} over a common time period of N years between t_0 and t_1:

$$\overline{\beta_{\mathrm{t,i}}} = \frac{M_{\mathrm{geod,i}} - \sum_{t_0}^{t_1} M_{\mathrm{glac}}}{N} \quad (5)$$

In a second step, we calculated the mean annual mass change ΔM_{ref} for t_{ref} for each individual glacier by adding $\overline{\beta_{\mathrm{t}}}$ to the mean annual glaciological mass balance for the reference period $M_{\mathrm{glac,t_{ref}}}$:

$$\Delta M_{\mathrm{t_{ref},i}} = M_{\mathrm{glac,t_{ref}}} + \beta_{\mathrm{t,i}} \quad (6)$$

For the homogenisation step, the series of Tuyuksu was used for Dzhungarsky Alatau, Western / Northern Tien Shan, Pamir-Alay and Western Pamir and the one of Urumqi for Eastern and Central Tien Shan and Eastern Pamir. All $\Delta M_{\mathrm{t_{ref},i}}$ were weighted according to their specific uncertainty and the median of all the weighted estimates was interpreted as the reference geodetic mass balance of the corresponding glacier (S1). This value was then used for a second-order model calibration. For each glacier, the calculated uncertainties of the mass changes obtained for the different periods are summarised through their arithmetic mean. This value is used to represent the uncertainty of the geodetic mass balance for the reference period.

Comparing homogenised geodetic mass balances with other studies

A glacier-by-glacier comparison of the mass balance with results provided in Brun, Berthier, Wagnon, Kääb, and Treichler (2017) agreed within the expected uncertainties with a mean difference of $-0.15\,\mathrm{m\,w.e.\,yr^{-1}}$, ours being more negative, and RMSE

of $0.27\,\mathrm{m\,w.e.\,yr^{-1}}$. Good agreement was obtained for the region-wide mean mass balance rate for the Tien Shan ($<-0.05\,\mathrm{m\,w.e.\,yr^{-1}}$). Region-wide differences were larger for the Pamir ($<-0.30\,\mathrm{m\,w.e.\,yr^{-1}}$). We compared the homogenised geodetic mass balances from 1999/00 to 2017/18 with the geodetic surveys published by Shean et al. (2020) and Hugonnet et al. (2021). We found good agreement for the Tien Shan (Fig. S2 and Table S1). However, geodetic mass balance estimates for the Pamir presented in this study were somewhat more negative than in Shean et al. (2020) and Hugonnet et al. (2021) (Fig. S2 and Table S1). Hugonnet et al. (2021) provided mass balance estimates covering the period 2000–2010 and 2010–2020 (Fig. S3). Their results show a clear negative shift with time for both the Tien Shan and the Pamir, with mass balance rates for the Pamir moving from near-balanced conditions (mean of all glaciers: $-0.03\pm 0.20\,\mathrm{m\,w.e.\,yr^{-1}}$) to slightly negative specific mass change rates ($-0.10\pm 0.20\,\mathrm{m\,w.e.\,yr^{-1}}$). For the Tien Shan, the glaciers showed clearly negative mass balance rates for both periods (mean of all glaciers, 2000–2010: $-0.26\pm 0.26\,\mathrm{m\,w.e.\,yr^{-1}}$ and 2010–2020: $-0.35\pm 0.22\,\mathrm{m\,w.e.\,yr^{-1}}$, Fig. S3). Our results for the Tien Shan agree reasonably well for both time periods. They are slightly less negative for the first period ($-0.19\pm 0.37\,\mathrm{m\,w.e.\,yr^{-1}}$) and become more negative thereafter (2010/11–2017/18: $-0.33\pm 0.37\,\mathrm{m\,w.e.\,yr^{-1}}$). For the Pamir, the results of the present study are substantially more negative for both periods than the geodetic estimates provided by Shean et al. (2020) and Hugonnet et al. (2021). For the same periods, we found a mass balance rates of $-0.25\pm 0.37\,\mathrm{m\,w.e.\,yr^{-1}}$ from 1999/00 to 2010/11 and of $-0.29\pm0.37\,\mathrm{m\,w.e.\,yr^{-1}}$ from 2010/11 to 2017/18 (Fig. S4). Overall, the homogenised geodetic mass balance for the Tien Shan is based on

image pairs well-distributed around 2010 (Fig. S5). For the Pamir, however, clearly more image pairs were available after 2010, when mass balances became increasingly negative (Fig. S5). Using the glaciological measurement time series to homogenise the individual geodetic surveys compensates for this. However, there is no continuous measurement series available for the Pamir. Due to this lack of data, we used the mass balance series of Tuyuksu Glacier, located in the North / Western Tien Shan (Fig. 1a) for homogenisation for the Western Pamir and the Pamir-Alay and Urumqi Glacier for the Eastern Pamir (see section "Homogenisation of geodetic mass balances" in Supplementary Matieral). Earlier studies showed that Tuyuksu has a similar mass balance as Abramov (lacking direct observations from 1998/99–2010/11) and other glaciers in the Pamir-Alay (Shchetinnikov, 1998). Our results clearly show a large mass balance heterogeneity in the Pamir and Abramov (2003–2014: -0.39 ± 0.16 m w.e. yr^{-1} Barandun et al. (2018)), Tuyuksu (1998–2016: -0.35 ± 0.18 m w.e. yr^{-1} Kapitsa et al. (2020)) and Urumqi (1981–2015: -0.46 ± 0.14 m w.e. yr^{-1}, Xu, Li, Li, Wang, and Zhou (2019)) are below the average glacier mass balances of the Western and Eastern Pamir. This might have influenced the homogenisation procedure for the region. However, all three geodetic studies used for comparison (Brun et al., 2017; Shean et al., 2020; Hugonnet et al., 2021) are mostly based on the same ASTER datasets. Independent mass change assessments for the Pamir are partly inconsistent however (Gardelle et al., 2013; Brun et al., 2017; Shean et al., 2020; Gardner et al., 2013; Kääb et al., 2015; Farinotti et al., 2015; Barandun et al., 2020), with our own estimate falling somewhere in between.

First-order model calibration

In a first step, we constrained the model parameters C_{prec} and DDF_{snow} for each glacier and year with SCAFs derived from the transient snowline observations. In Barandun et al. (2018), an initial range for DDF_{snow} and C_{prec} was narrowed down until an optimal parameter combination was obtained. In order to limit computation time, the calibration procedure was adjusted for a regional application (Fig. S6).

C_{prec} constrained the modelled accumulation of a specific year and glacier. It was adjusted to minimise the root mean square error (RMSE) between the modelled cumulative melt $Melt_{\mathrm{modelled}}$ at the position of the observed snowlines, and the total amount of accumulated winter snow $Accu_{\mathrm{modelled}}$ that melted from the onset of the ablation season until the snowline observation date (Fig. S7, Barandun et al. (2018)). DDF_{snow} was calibrated to best represent all SCAF observations of one ablation season by reducing the RMSE between modelled and observed SCAF $RMSE_{\mathrm{SCAF}}$ (Barandun et al., 2018). C_{prec} and DDF_{snow} were calibrated annually and for each glacier separately to correctly represent the winter snow accumulation and the melt rates for each year of the observation period. More details are given in (Barandun et al., 2018).

We do not explicitly describe internal and basal balance and sublimation but account for them within the expected uncertainties by integrating geodetic mass balances into the calibration procedure. As a control, the mountain range mass balance of both methods (transient snowline constrained mass balance reconstruction / geodetic method) was compared and no systematic offset (mean absolute error = $+0.09\,\mathrm{m\ w.e.\,yr^{-1}}$) was found

(correlation coefficient = 0.71; RMSE = 0.28 m w.e. yr^{-1}).

Limitations

The applicability of the transient snowline-constrained modelling is primarily depending on the representativeness of the transient snowline for the transient surface mass balance. Thus, the model is in principal applicable on glaciers for which a clear relationship between the transient snowline and the surface mass balance exists. Such a relationship is found for most winter-accumulation type glaciers and might be questionable for certain cases i.e. (i) presence of superimposed ice, (ii) summer snowfall, (iii) non-winter-accumulation type glaciers, (iv) debris covered, (v) surge-type glaciers and (vi) glaciers with important contribution of internal and basal balances. The here applied methodology however does not depend on a single image but a set of seasonally distributed snowline observations throughout the ablation season, so that the effect of (i) and (ii) can be reduced. We use geodetic estimates for model calibration to limit the effect of (iv), (v) and (vi). Summer accumulation and all year round ablation are not taken in account for calibration, but they are included in the modelled daily mass balance. Therefore, it allows quantifying the annual mass balance for (iii) but makes the interpretation of seasonal to sub-seasonal mass balance time series more uncertain.

Debris cover

The influence of debris cover on glacier mass balance is complex and obscures the climate-related response of glacier mass changes. The approach applied here does not ac-

count for debris cover, and thus might over- or underestimate melt rates of the underlying ice. However, the sub-seasonal transient snowline variation still reflects the annual variability of the surface mass balance. The model is constrained to match decadal geodetic mass changes. Computed decadal mass changes thus agree with observations (inherently accounting for the effect of a debris layer at the surface as well as internal and basal mass change). Hence, through the applied second-order calibration, the reproduced mass balance is as accurate as the geodetic estimate. The annual mass balance variability is as accurate as the snowline-constrained annual time series. Brun et al. (2019) reported that the glacier mass balance has a significant positive correlation with debris cover for the Tien Shan but not for the Pamir. The authors further found a negative correlation between the two quantities for the Pamir-Alay and state the limited explanatory power of supraglacial debris for reported decadal mass balances of High Mountain Asia.

Glacier outlines

The glacier mass balance depends upon both, the climate and the configuration of the glacier geometry, each of which is a continuous function of time (Elsberg et al., 2001). The so-called "reference" mass balance removes the surface change effects by holding the glacier surface constant through time, and thus is the climatically relevant mass balance. However, it does not precisely quantify the actual annual mass gain and loss for a non-updated outline. The so-called "conventional" mass balance takes in account the glacier geometry change and is thus relevant for quantifying meltwater release during a mass balance year (Harrison et al., 2005). Here, we chose constant glacier outlines taken from RGIv6.0 to estimate the reference mass balance. Most of the RGIv6.0 outlines for Central

Asia date from the onset of the century and therefore give a good basis to calculate the reference mass balance from 1999/00 to 2017/18. Some regions (Eastern Pamir, Eastern Tien Shan, Dzungarsky Alatau and the Eastern part of the Central Tien Shan) contain outlines not only from the beginning of the study period but also from 2007 and 2009. The choice of the reference surface affects the reference mass balance. It has been shown that the trend in the difference between the glacier-wide cumulative and the reference mass balance will grow approximately linearly with time but the difference between the glacier-wide annual balances on two different reference surfaces will be approximately constant over the years (Elsberg et al., 2001). For South Cascade glaciers, a difference in mass balance of 16% for the period 1970 to 1997 between conventional and reference balance was found (Elsberg et al., 2001). Following evolution, the difference in the mass balances using outlines from 2000 and 2009, respectively, would result in a difference of mass balance of less then 5%. The cumulative mass balance however is more sensitive to the choice of the reference area (Elsberg et al., 2001).

Model calibration

The model calibration is based on the information content of the transient snowline observation. In order to apply the approach on glaciers without direct measurements, the use of additional ground-based information was avoided. Thus, the two calibrated parameters are not strictly independent; meaning that an overestimated accumulation rate can be compensated by a too high ablation rate without degrading the agreement with the transient snowline observations. Erroneous seasonal components do not affect

excessively the calculated annual balance as they equal out but introduce considerable uncertainties for the seasonal to sub-seasonal model outputs. An attempt to overcome this problem was made by iterative calibration, where both parameters were adjusted in parallel (Barandun et al., 2018). With this iterative calibration, the model tends towards an optimal solution for both parameters without the need of additional observations. Because winter measurements are sparse for the region a detailed validation of the seasonal components was so far not possible, however, for the few years and few glaciers for which winter measurements are available, the calibration procedures provided satisfying results (Barandun et al., 2018). Nonetheless, at the current stage of the work, interpretation of seasonal to sub-seasonal mass balance time series has largely been avoided and more work is needed to properly evaluate the model performance on increased temporal resolution.

References

Barandun, M., Fiddes, J., Scherler, M., Mathys, T., Saks, T., Petrakov, D., & Hoelzle, M. (2020). The state and future of the cryosphere in Central Asia. *Water Security*, *11*, 100072. doi: https://doi.org/10.1016/j.wasec.2020.100072

Barandun, M., Huss, M., Sold, L., Farinotti, D., Azisov, E., Salzmann, N., ... Hoelzle, M. (2015). Re-analysis of seasonal mass balance at abramov glacier 1968–2014. *Journal of Glaciology*, *61*(230), 1103–1117.

Barandun, M., Huss, M., Usubaliev, R., Azisov, E., Berthier, E., Kääb, A., ... Hoelzle, M. (2018). Multi-decadal mass balance series of three Kyrgyz glaciers inferred from modelling constrained with repeated snow line observations. *The Cryosphere*, *12*(6), 1899–1919. doi: 10.5194/tc-12-1899-2018

Bolch, T., Shea, J. M., Liu, S., Azam, F. M., Gao, Y., Gruber, S., ... others (2019). Status and change of the cryosphere in the Extended Hindu Kush Himalaya Region. In *The Hindu Kush Himalaya Assessment* (pp. 209–255). Springer. doi: 10.1007/978-3-319-92288-1_7

Brun, F., Berthier, E., Wagnon, P., Kääb, A., & Treichler, D. (2017). A spatially resolved estimate of High Mountain Asia glacier mass balances, 2000-2016. *Nature Geoscience*, *10*(9), 668. doi: 10.1038/ngeo2999

Brun, F., Wagnon, P., Berthier, E., Jomelli, V., Maharjan, S., Shrestha, F., & Kraaijenbrink, P. (2019). Heterogeneous influence of glacier morphology on the mass balance variability in High Mountain Asia. *Journal of Geophysical Research: Earth Surface*, *124*(6), 1331–1345. doi: 10.1029/2018JF004838

Claverie, M., Vermote, E. F., Franch, B., & Masek, J. G. (2015). Evaluation of the Landsat-5 TM and Landsat-7 ETM+ surface reflectance products. *Remote Sensing of Environment*, *169*, 390–403. doi: 10.1016/j.rse.2015.08.030

Cuffey, K., & Paterson, W. (2010). *The physics of glaciers* (forth ed.). Oxford: Butterworth-Heinemann. (pp. 704) doi: 10.14430/arctic2477

Elsberg, D., Harrison, W., Echelmeyer, K., & Krimmel, R. (2001). Quantifying the effects of climate and surface change on glacier mass balance. *Journal of glaciology*, *47*(159), 649–658. doi: 10.3189/172756501781831783

Farinotti, D., Longuevergne, L., Moholdt, G., Duethmann, D., Mölg, T., Bolch, T., ... Güntner, A. (2015). Substantial glacier mass loss in the Tien Shan over the past 50 years. *Nature Geoscience*, *8*(9), 716–722. doi: 10.1038/ngeo2513

Gardelle, J., Berthier, E., Arnaud, Y., & Kääb, A. (2013). Region-wide glacier mass balances over the Pamir-Karakoram-Himalaya during 1999–2011. *The Cryosphere*, *7*(4), 1263–1286. doi: 10.5194/tc-7-1263-2013

Gardner, A., Moholdt, G., Cogley, J., Wouters, B., Arendt, A., Wahr, J., ... Paul, F. (2013). A Reconciled Estimate of Glacier Contributions to Sea Level Rise: 2003 to 2009. *Science*, *340*, 852-857. doi: 10.1126/science.1234532

Girod, L., Nuth, C., Kääb, A., McNabb, R., & Galland, O. (2017). MMASTER: Improved ASTER DEMs for Elevation Change Monitoring. *Remote Sensing*, *9*(7), 704. doi: 10.3390/rs9070704

Hall, D., Ormsby, J., Bindschadler, R., & Siddalingaiah, H. (1987). Characterization of snow and ice reflectance zones on glaciers using Landsat Thematic Mapper data.

Annals of Glaciology, *9*, 104–108.

Harrison, W., Elsberg, D., Cox, L., & March, R. (2005). Different mass balances for climatic and hydrologic applications. *Journal of Glaciology*, *51*(172), 176–176. doi: 10.3189/172756505781829601

Hugonnet, R., McNabb, R., Berthier, E., Menounos, B., Nuth, C., Girod, L., ... Kääb, A. (2021). A globally complete, spatiotemporally resolved estimate of glacier mass change from 2000 to 2019. *Nature*. doi: 10.1038/s41586-021-03436-z

Huss, M. (2013). Density assumptions for converting geodetic glacier volume change to mass change. *The Cryosphere*, *7*(3), 877–887. doi: 10.5194/tc-7-877-2013

Kääb, A., Treichler, D., Nuth, C., & Berthier, E. (2015). Brief Communication: Contending estimates of 2003–2008 glacier mass balance over the Pamir–Karakoram–Himalaya. *The Cryosphere*, *9*(2), 557–564. doi: 10.5194/tc-9-557-2015

Kapitsa, V., Shahgedanova, M., Severskiy, I., Kasatkin, N., White, K., & Usmanova, Z. (2020). Assessment of changes in mass balance of the tuyuksu group of glaciers, northern tien shan, between 1958 and 2016 using ground-based observations and pléiades satellite imagery. *Frontiers in Earth Science*, *8*, 259.

Kronenberg, M., Barandun, M., Hoelzle, M., Huss, M., Farinotti, D., Azisov, E., ... Kääb, A. (2016). Mass-balance reconstruction for glacier no. 354, tien shan, from 2003 to 2014. *Annals of Glaciology*, *57*(71), 92–102.

Kruse, F. A., Lefkoff, A., Boardman, J., Heidebrecht, K., Shapiro, A., Barloon, P., & Goetz, A. (1993). The spectral image processing system (SIPS)—interactive visualization and analysis of imaging spectrometer data. *Remote sensing of environment*,

44(2-3), 145–163. doi: 10.1016/0034-4257(93)90013-N

Liang, S. (2001). Narrowband to broadband conversions of land surface albedo I: Algorithms. *Remote Sensing of Environment*, *76*(2), 213–238. doi: 10.1016/S0034-4257(00)00205-4

Masek, J. G., Vermote, E. F., Saleous, N. E., Wolfe, R., Hall, F. G., Huemmrich, K. F., ... Lim, T.-K. (2006). A Landsat surface reflectance dataset for North America, 1990-2000. *IEEE Geoscience and Remote Sensing Letters*, *3*(1), 68–72. doi: 10.1109/LGRS.2005.857030

Maussion, F., Scherer, D., Mölg, T., Collier, E., Curio, J., & Finkelnburg, R. (2014). Precipitation seasonality and variability over the Tibetan Plateau as resolved by the High Asia Reanalysis. *Journal of Climate*, *27*(5), 1910–1927. doi: 10.1175/JCLI-D-13-00282.1

McNabb, R. W., Nuth, C., Kääb, A., & Girod, L. (2019). Sensitivity of glacier volume change estimation to DEM void interpolation. *The Cryosphere*, *13*, 895–910. doi: 10.5194/tc-13-895-2019

Naegeli, K., Damm, A., Huss, M., Wulf, H., Schaepman, M., & Hoelzle, M. (2017). Cross-Comparison of albedo products for glacier surfaces derived from airborne and satellite (Sentinel-2 and Landsat 8) optical data. *Remote Sensing*, *9*(2), 110. doi: 10.3390/rs90201010

Naegeli, K., & Huss, M. (2017). Sensitivity of mountain glacier mass balance to changes in bare-ice albedo. *Annals of Glaciology*, 1–11. doi: 10.1017/aog.2017.25

Naegeli, K., Huss, M., & Hoelzle, M. (2019). Change detection of bare-ice albedo in the

Swiss Alps. *The Cryosphere*, *13*(1), 397–412. doi: 10.5194/tc-13-397-2019

Pieczonka, T., & Bolch, T. (2015). Region-wide glacier mass budgets and area changes for the Central Tien Shan between ∼1975 and 1999 using Hexagon KH-9 imagery. *Global and Planetary Change*, *128*, 1–13. doi: 10.1016/j.gloplacha.2014.11.014

RGI Consortium. (2017). *Randolph Glacier Inventory – A Dataset of Global Glacier Outlines: Version 6.0: Technical Report.* Global Land Ice Measurements from Space, Colorado, USA. doi: 10.7265/N5-RGI-60

Shchetinnikov, S. (1998). *Morphologiya i regime lednikov pamiroalaya [the morphology and regime of pamir-alai glaciers].*

Shean, D., Bhushan, S., Montesano, P., Rounce, D. R., Arendt, A., & Osmanoğlu, B. (2020). A systematic, regional assessment of high mountain Asia glacier mass balance. *Frontiers in Earth Science*, *7*, 363. doi: 10.3389/feart.2019.00363

Vermote, E., Justice, C., Claverie, M., & Franch, B. (2016). Preliminary analysis of the performance of the Landsat 8/OLI land surface reflectance product. *Remote Sensing of Environment*, *185*, 46–56. doi: 10.1016/j.rse.2016.04.008

WGMS. (2017). *Global Glacier Change Bulletin No. 2 (2014-2015)* (Vol. 2; M. Zemp et al., Eds.). Zürich: ICSU (WDS) / IUGG(IACS) / UNEP / UNESCO / WMO, World Glacier Monitoring Service. doi: doi:10.5904/wgms-fog-2017-10

Xu, C., Li, Z., Li, H., Wang, F., & Zhou, P. (2019). Long-range terrestrial laser scanning measurements of annual and intra-annual mass balances for urumqi glacier no. 1, eastern tien shan, china. *The Cryosphere*, *13*(9), 2361–2383.

Zemp, M., Huss, M., Thibert, E., Eckert, N., McNabb, R., Huber, J., . . . others (2019).

Global glacier mass changes and their contributions to sea-level rise from 1961 to 2016. *Nature*, *568*(7752), 382–386. doi: 10.1038/s41586-019-1071-0

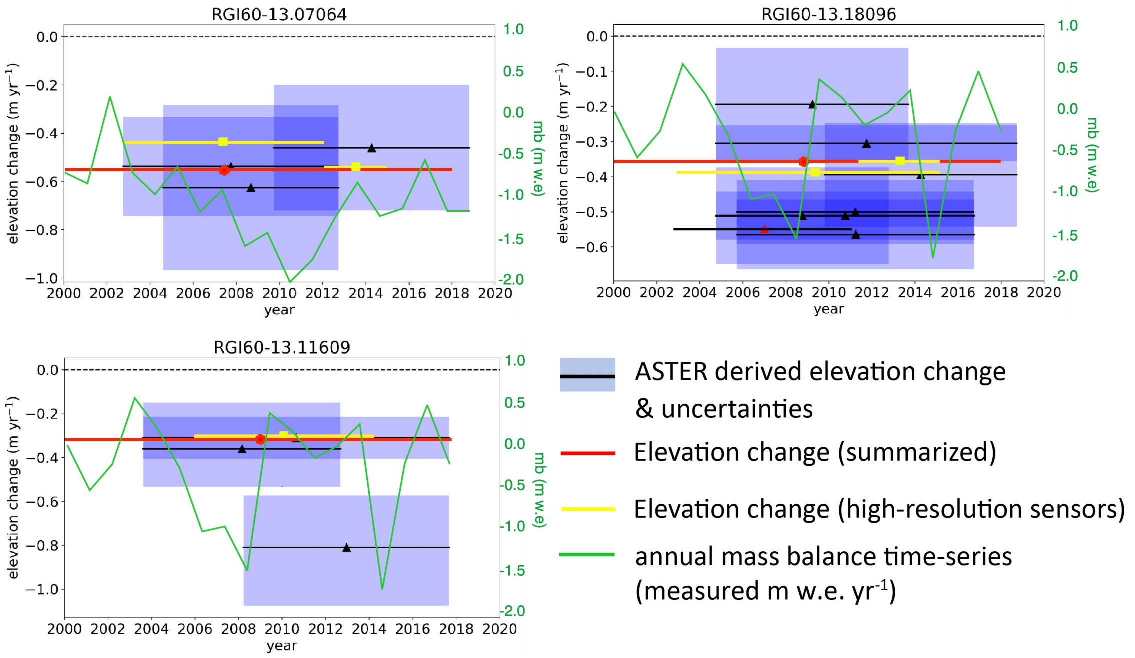

Figure S1. Example of geodetic mass balance for three selected glaciers in comparison to elevation change derived from high-resolution datasets. In red the geodetic mass balanced for the reference period 1999/00 to 2017/18 that takes into account all available geodetic mass balances per glacier, is indicated. The mass balances are homogenised using annual time series of glaciological measurements (green).

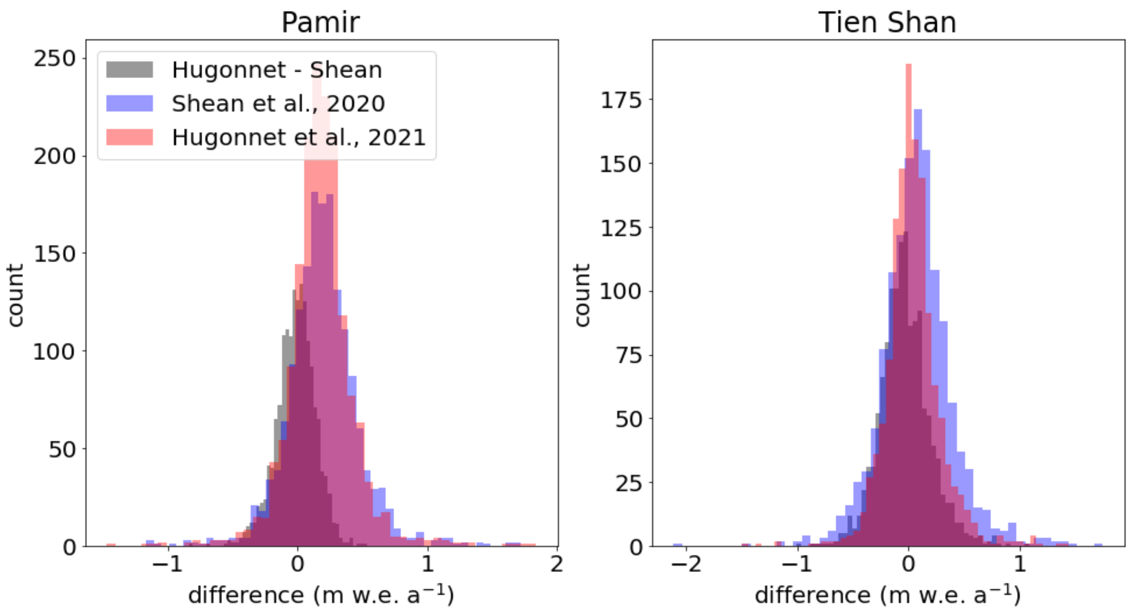

Figure S2. Comparison between the geodetic estimates of different studies for the Tien Shan and Pamir.

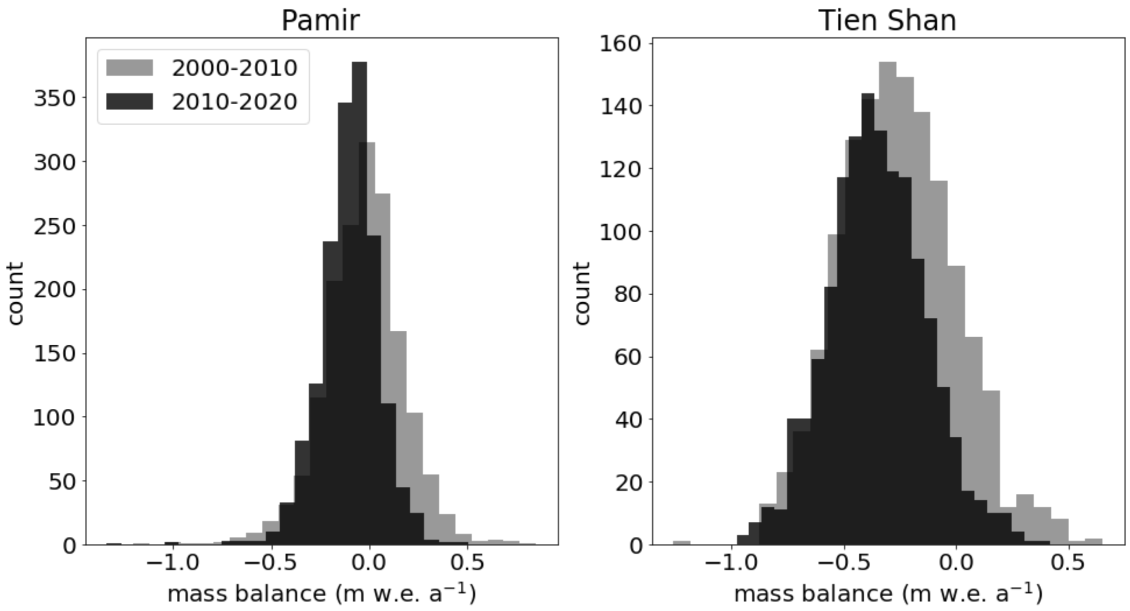

Figure S3. Comparison of the geodetic mass balances published in Hugonnet et al. (2021) for the Tien Shan and Pamir for the period 2000 to 2010 and 2011 to 2020.

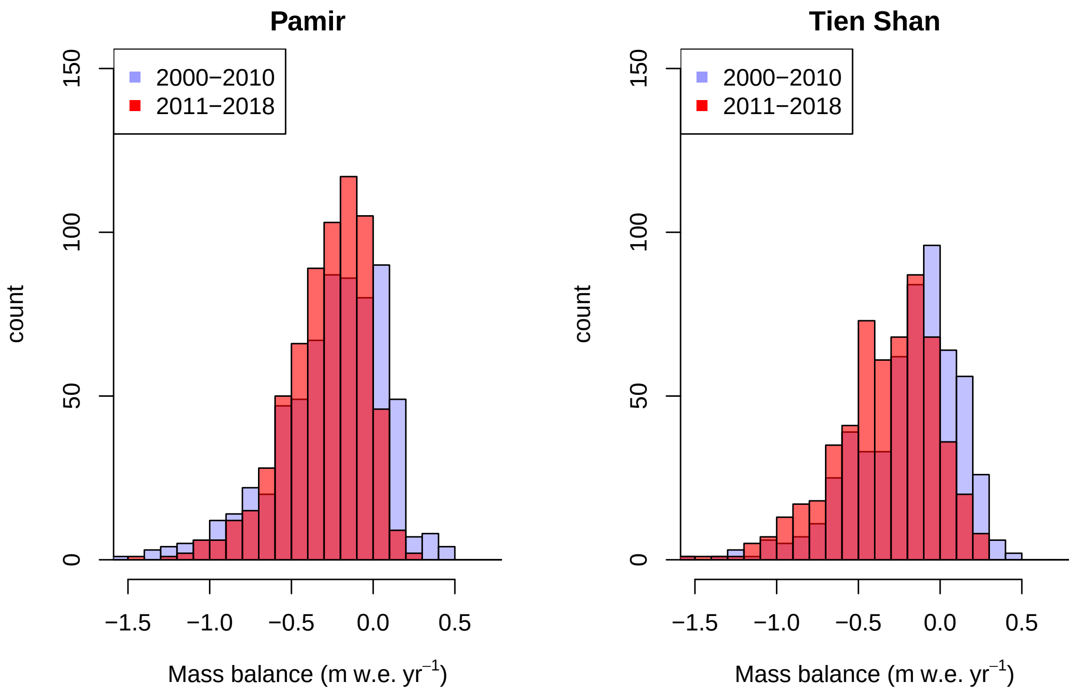

Figure S4. Comparison of the modelled (this study) mass balances for the Tien Shan and Pamir for the period 1999/10 to 2009/10 and 2010/11 to 2017/18

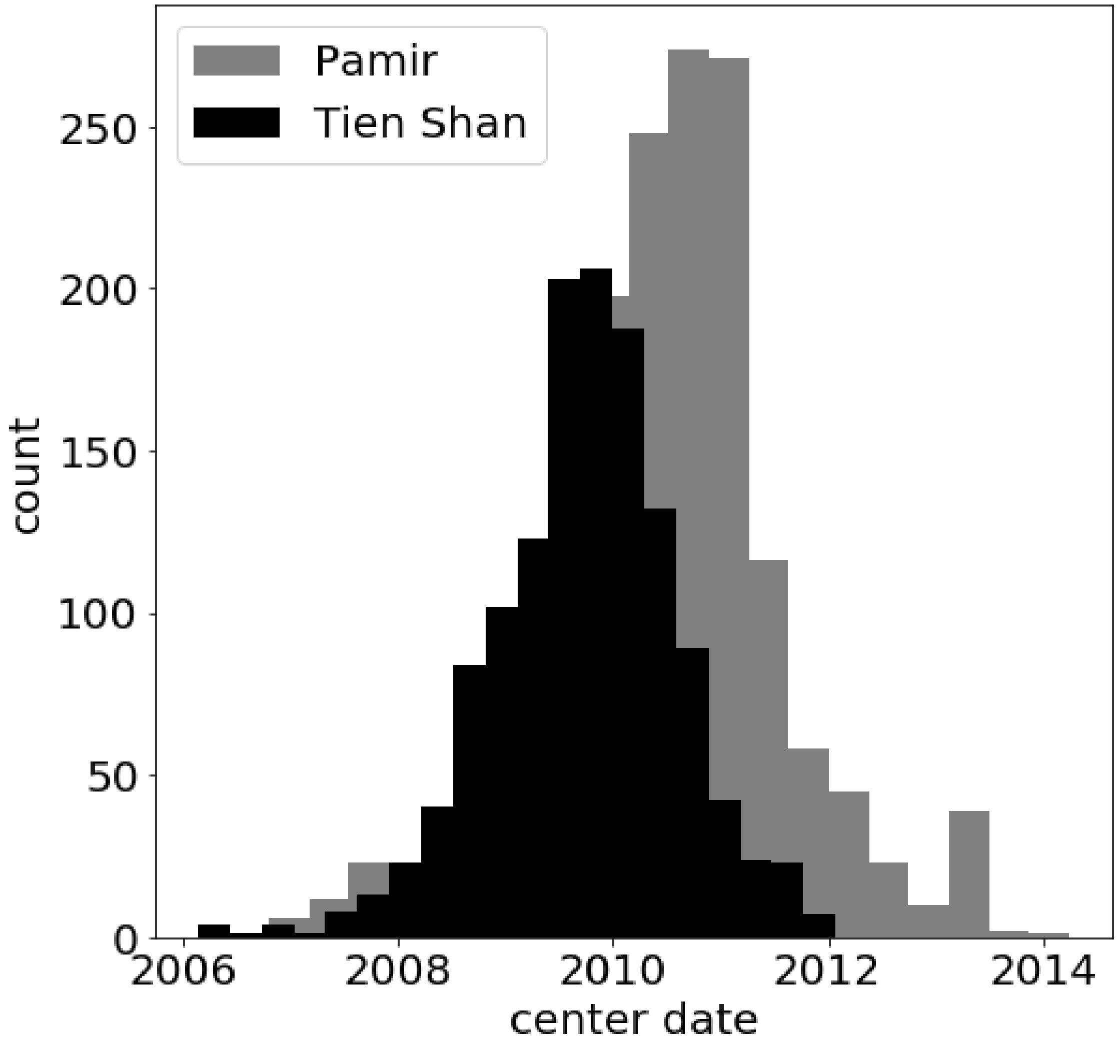

Figure S5. Numbers of image pairs according to their center date available to homogenise the geodetic mass balances for the Tien Shan and Pamir.

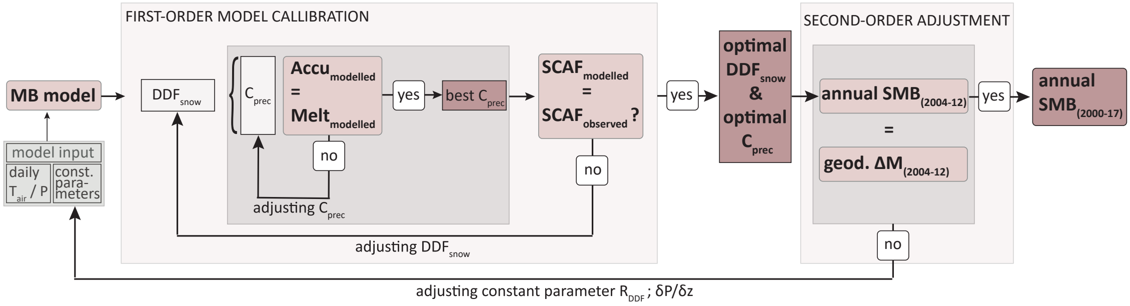

Figure S6. Calibration procedure to obtain an ideal combination of DDF_{snow} and C_{prec}. In a first step, DDF_{snow} and C_{prec} are optimised through comparison to snowline observations until a good solution for both parameters was found. For the initial value of DDF_{snow}, the best value of C_{prec} was determined constraining the modelled cumulative melt $Melt_{\mathrm{modelled}}$ at the snowline position to agree with the modelled winter snow accumulation $Accu_{\mathrm{modelled}}$ for the same location. Then, the model performance was evaluated to minimise $RMSE_{\mathrm{SCAF}}$ of the modelled and observed snow-covered area fractions (SCAF). This is repeated until no improvement in RMSE is obtained. In a second step, the modelled mass balance from 2004 to 2012 is compared to the multi-annual geodetic mass balance, and the relation between the degree day factor of snow and ice (R_{DDF}) and the precipitation gradient $\delta P/\delta z$ were readjusted until the results of both approaches agree within the uncertainty range of the geodetic estimate.

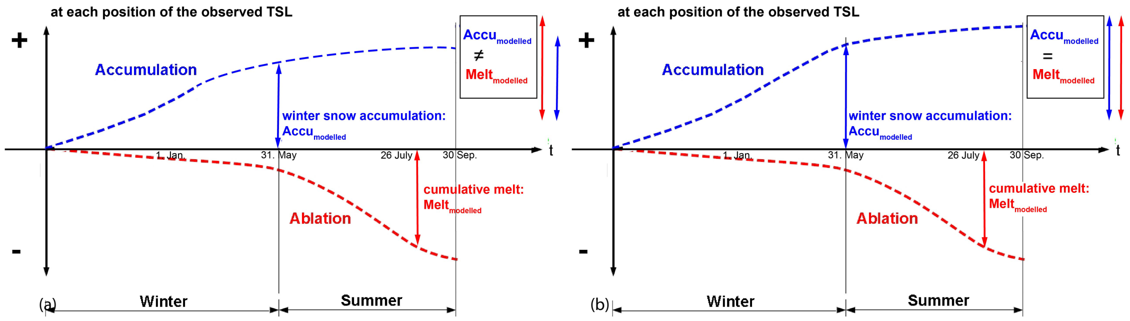

Figure S7. Calibration procedure for C_{prec}. The mass balance model is initially run with a preset of model parameters based on Barandun et al. (2018). For each snowline observation, the modelled melt along the mapped snowline is compared to the total accumulation that was modelled at this position. (a) gives an example for a snowline observation of the 26th of July. After this initial run, $Melt_{\text{modelled}}$ does not agree with $Accu_{\text{modelled}}$. C_{prec} is thus adjusted iteratively until (b) best possible agreement is reached, considering all snowline observations of one glacier throughout one summer season.

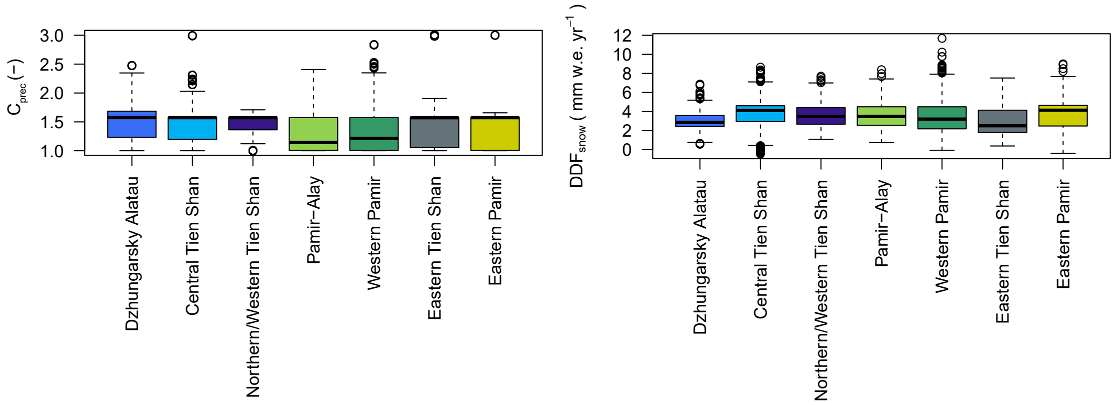

Figure S8. Box plots show parameters per year and per glacier summarised for the subregions for (A) C_{prec} and (B) DDF_{snow}

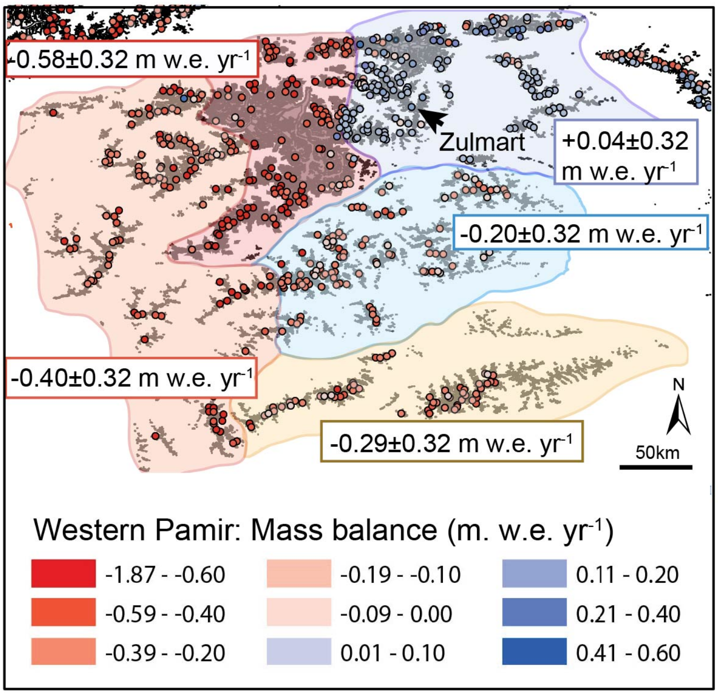

Figure S9. Mean mass balance from 1999/00 to 2017/18 for all considered glaciers (dots) of the Western Pamir. The coloured fields indicate regions of similar mass balance based on the here presented results. For each subregion, the averaged mass balances from 1999/00 to 2017/18 are given in boxes. Zulmart glacier is a glacier with glaciological measurements since 2018 located in the Western Pamir and part of a long-term monitoring programme. Based on our results, this glacier represents only a small part of the regional glacier mass balances of the Western Pamir.

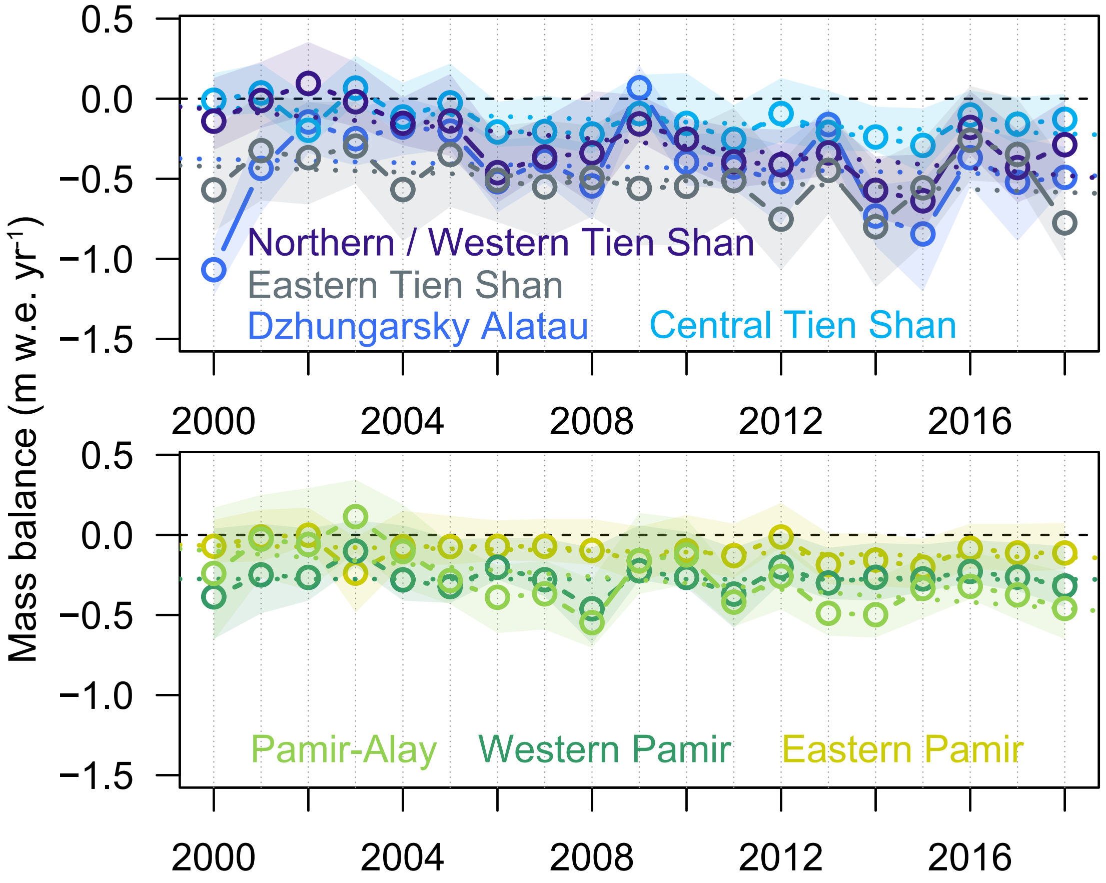

Figure S10. Mean annual mass balance time series for each subregion of the Tien Shan (blue) and the Pamir (green) from 1999/00 to 2017/18.

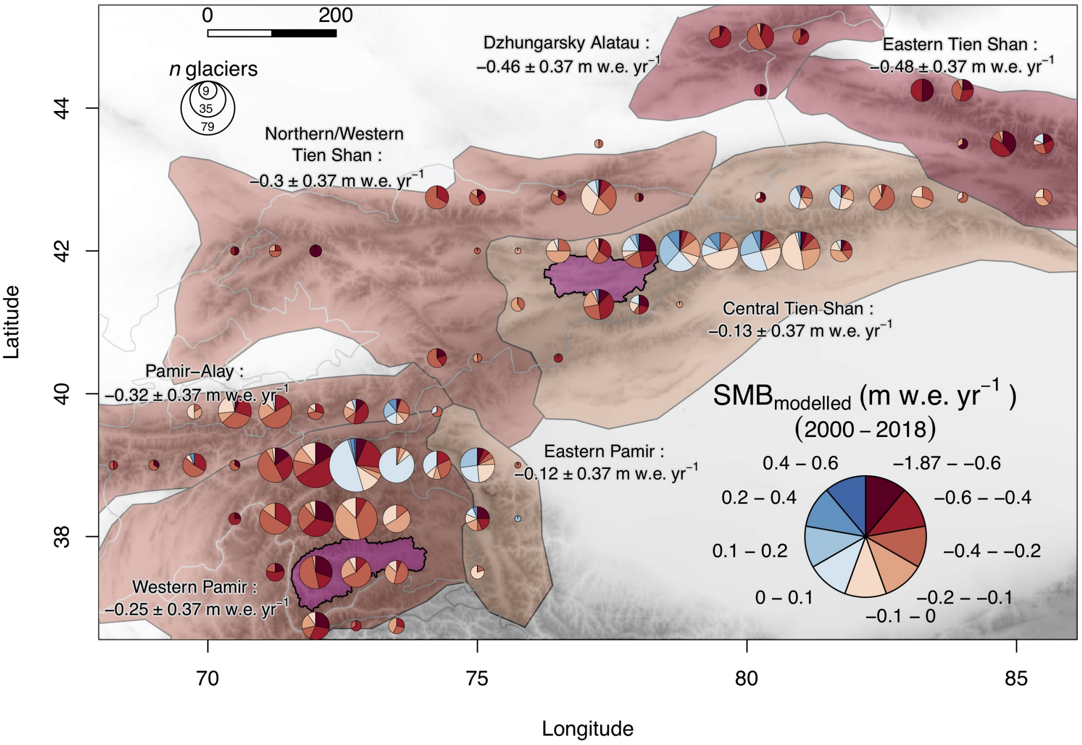

Figure S11. As Fig.1 (main text) but with different aggregation: Mean annual mass balances from 1999/00 to 2017/18 for the different sub-regions used in this study. Pie slice sizes represent percentage of glaciers that fall into a given mass balance category (aggregated to 0.75 degree grid cells corresponding to the ERA-Interim meteorological input dataset). Regional mass balances (text) are area-weighted means of individual glacier values. Strong spatial mass balance variability per aggregated grid cell showcase no direct dependency on the coarse input dataset used.

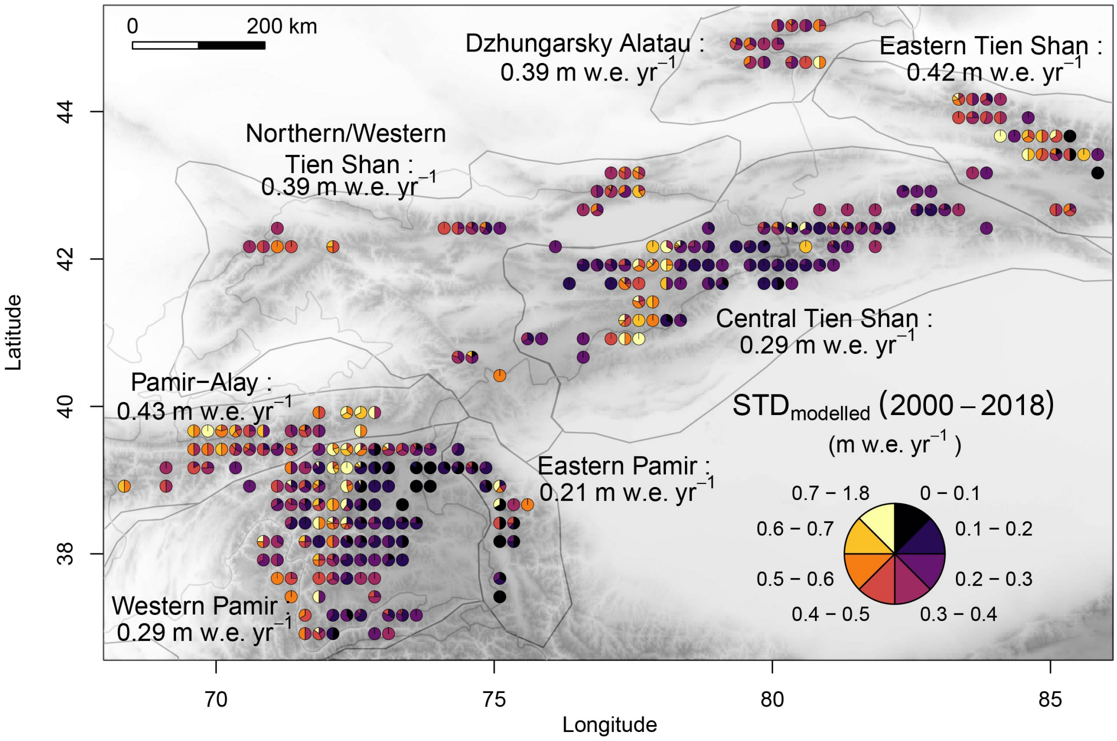

Figure S12. Year-to-year variability by means of standard deviation (σ) of mass balance from 1999/00 to 2017/18 of each individual glacier aggregated for 0.25 degree grid cells according to the glacier polygon centroids. Numbers indicate the mean σ for each subregion. Pie slice sizes represent percentage of glaciers that fall into a certain σ category. Pie size are not scaled to total number of glaciers per pixel, which can vary significantly.

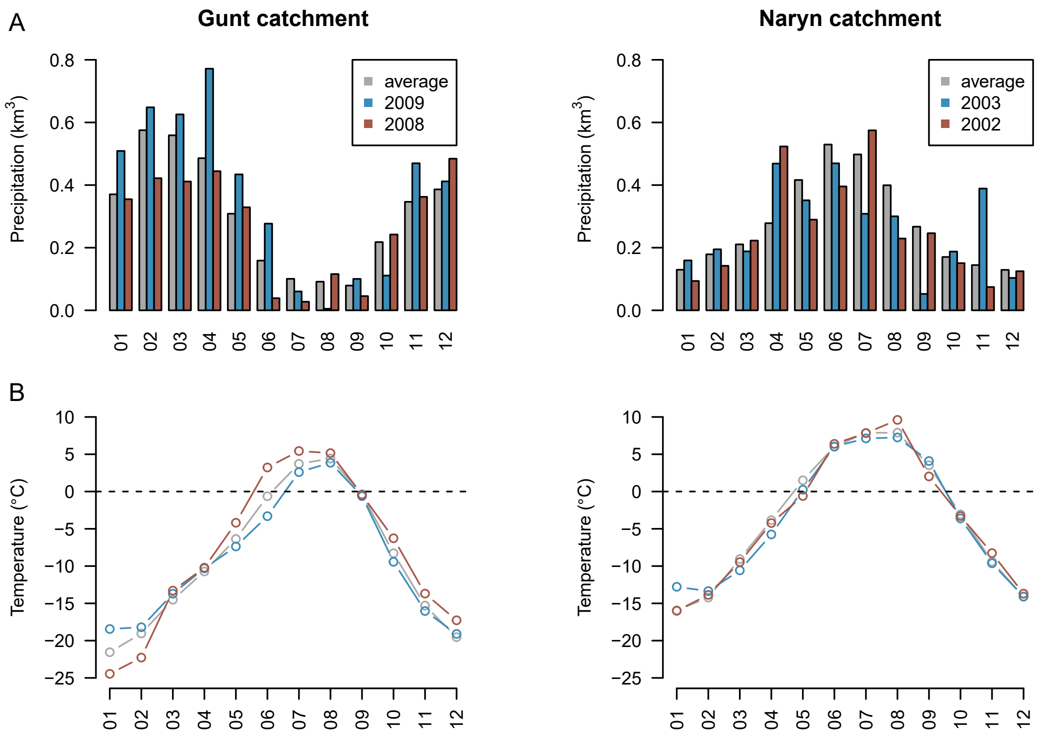

Figure S13. In order to investigate possible relationships of the mass balance with the key meteorological drivers, precipitation and temperature, we used the independent dataset High Asia Refined analysis (HAR) version 1.4 (Maussion et al., 2014). **A**. Monthly precipitation from HAR30 Reanalysis for the two catchments, for a below-average (red), and an above-average (red) mass balance year. **B**. Same but for monthly temperature.

Table S1. Comparison of modelled mass balances with geodetic estimates of other studies (Shean et al., 2020; Hugonnet et al., 2021). Mass balances of this study are mean area-weighted values for the period 1999/00 to 2017/18. Comparison data are geodetic mass balances from 2000 to 2020 ((Hugonnet et al., 2021)) and 2000 to 2018 ((Shean et al., 2020)).

	Tien Shan	Pamir
Shean et al. (2020) vs. this study	$0.07\pm 0.33\,\mathrm{m\,w.e.\,yr^{-1}}$	$0.19\pm 0.29\,\mathrm{m\,w.e.\,yr^{-1}}$
Hugonnet et al. (2021) vs. this study	$0.04\pm 0.25\,\mathrm{m\,w.e.\,yr^{-1}}$	$0.18\pm 0.26\,\mathrm{m\,w.e.\,yr^{-1}}$
Hugonnet et al. (2021) vs. Shean et al. (2020)	$-0.03\pm 0.23\,\mathrm{m\,w.e.\,yr^{-1}}$	$-0.01\pm 0.15\,\mathrm{m\,w.e.\,yr^{-1}}$

Table S2. Constant model parameters used in Barandun et al. (2018) were adopted for the initiation of the model. The mean parameters for the precipitation correction C_{prec} and DDF DDF_{snow} obtained for modelling are also given.

$\delta T/\delta z$	-4.8	$^{\circ}\mathrm{C\,km^{-1}}$
$\delta P/\delta z$	6.4	$10^{-4}\,\mathrm{m^{-1}}$
R_{DDF}	1.57	–
DDF_{snow}	3.37 ± 1.4	$\mathrm{mm\,day^{-1}\,^{\circ}C^{-1}}$
C_{prec}	1.25 ± 0.6	–

Table S3. Standard deviation (STD) of the mass balance time series of Golubin, Abramov and Glacier No. 354. The standard deviation represents the year-to-year mass balance variability of the time series. STD(meas) is the standard deviation purely based on direct measurements (WGMS, 2017). The STD (conventional model) refers to the standard deviation of the mass balance time series based on a mass balance model presented in Barandun et al. (2015) and Kronenberg et al. (2016) that uses a few years of direct observations to calibrate a mass balance model to reconstruct mass balances for years without measurements. STD(TSL-model) refers to the standard deviation based on modelled mass balances, constrained with transient snowline observations.

glacier	STD (meas)	STD (conventional model)	STD (TSL-model)
Golubin	$0.31\,\mathrm{m\,w.e.\,yr^{-1}}$	$0.58\,\mathrm{m\,w.e.\,yr^{-1}}$	$0.41\,\mathrm{m\,w.e.\,yr^{-1}}$
Abramov	$0.28\,\mathrm{m\,w.e.\,yr^{-1}}$	$0.53\,\mathrm{m\,w.e.\,yr^{-1}}$	$0.31\,\mathrm{m\,w.e.\,yr^{-1}}$
No. 354	$0.09\,\mathrm{m\,w.e.\,yr^{-1}}$	$0.30\,\mathrm{m\,w.e.\,yr^{-1}}$	$0.14\,\mathrm{m\,w.e.\,yr^{-1}}$

Table S4. Monthly discharge (m^3s^{-1}) of the Gunt and Naryn Rivers as average, and for extremely negative and positive mass balance years obtained through Tajik Hydromet Office and Central-Asian Institute for Applied Geosciences. Baseline for the calculation of monthly means is the period 1999/00 to 2012/13 (Gunt) and 1999/00 to 2017/18 (Naryn), respectively.

	Gunt			Naryn		
Year	Avg.	2009	2008	Avg.	2003	2006
Jan	39.8	40.5	48.2	31.4	32.2	30.4
Feb	36.3	36.7	45.7	29.5	31.8	27.8
Mar	34.3	34.9	41.6	30.9	33.1	28.9
Apr	36.2	31.1	33.6	60.6	44.0	62.6
May	81.6	40.8	97.6	143.0	181.0	153.0
Jun	220.8	136.7	222.6	246.1	360.0	173.0
Jul	279.8	341.0	194.9	249.3	268.0	229.0
Aug	208.6	248.9	204.6	215.7	194.0	247.0
Sep	111.6	93.0	102.3	108.9	133.0	100.0
Oct	63.9	60.5	55.6	62.3	64.0	56.2
Nov	49.3	50.5	39.3	43.3	48.7	39.0
Dec	44.4	45.9	42.9	33.5	39.1	30.0

Table S5. Total area, amount and median elevation of all glaciers, and number of studied glaciers >2 km^2 (based on RGIv6.0 (RGI Consortium, 2017)) for the subregions of Tien Shan (TS) and Pamir (Bolch et al., 2019). The resulting unweighted mean equilibrium line altitude (ELA) and accumulation area ratio (AAR) from 1999/00 to 2017/18 for the specific subregions are given.

Study regions	Glacierised area (km^2)	No. glaciers	Median elev. (m a.s.l.)	No. mod. glaciers	$ELA_{(2000-18)}$ (m a.s.l.)	$AAR_{(2000-18)}$ (%)
Northern / Western TS	1192	108	3864	94	4009	35
Eastern Tien Shan	2333	114	3973	66	3803	66
Central Tien Shan	8340	577	4155	357	4121	60
Dzhungarsky Alatau	521	50	3610	46	3753	32
Pamir-Alay	3151	151	4064	108	4184	39
Western Pamir	8107	685	4841	475	5170	49
Eastern Pamir	1750	156	5045	76	5119	62
Tien Shan	12385	1000	4023	563	4026	56
Pamir / Pamir-Alay	12080	993	4686	659	4995	48
all	24465	1993	4336	1222	4530	52
